# LncRNA Snhg1-driven self-reinforcing regulatory network promoted cardiac regeneration and repair after myocardial infarction

**DOI:** 10.7150/thno.57037

**Published:** 2021-09-13

**Authors:** Mengsha Li, Hao zheng, Yuan Han, Yijin Chen, Bing Li, Guojun Chen, Xiaoqiang Chen, Senlin Huang, Xiang He, Guoquan Wei, Tong Xu, Xiaofei Feng, Wangjun Liao, Yulin Liao, Yanmei Chen, Jianping Bin

**Affiliations:** 1 Department of Cardiology and National Key Lab for Organ Failure Research, Nanfang Hospital, Southern Medical University, Guangzhou, 510515, China.; 2 Bioland Laboratory (Guangzhou Regenerative Medicine and Health Guangdong Laboratory), Guangzhou 510005, China.; 3 Guangdong Provincial Key Laboratory of Shock and Microcirculation, Nanfang Hospital, Southern Medical University, Guangzhou 510515, China.; 4 School of Medicine, Guizhou University, Guiyang, Guizhou, 550025, China.; 5 Department of Oncology, Nanfang Hospital, Southern Medical University, Guangzhou, 510515, China.

**Keywords:** Myocardial infarction, Cardiac regeneration, feedback loops, Snhg1, long non-coding RNA

## Abstract

**Rationale:** Most current cardiac regeneration approaches result in very limited cell division and little new cardiomyocyte (CM) mass. Positive feedback loops are vital for cell division, but their role in CM regeneration remains unclear. We aimed to determine whether the lncRNA small nucleolar RNA host gene 1 Snhg1 (Snhg1) could form a positive feedback loop with c-Myc to induce cardiac regeneration.

**Methods:** Quantitative PCR and *in situ* hybridization experiments were performed to determine the Snhg1 expression patterns in fetal and myocardial infarction (MI) hearts. Gain- and Loss-of-function assays were conducted to explore the effect of Snhg1 on cardiomyocyte (CM) proliferation and cardiac repair following MI. We further constructed CM-specific Snhg1 knockout mice to confirm the proliferative effect exerted by Snhg1 using CRISPR/Cas9 technology. RNA sequencing and RNA pulldown were performed to explore how Snhg1 mediated cardiac regeneration. Chromatin immunoprecipitation and luciferase reporter assays were used to demonstrate the positive feedback loop between Snhg1 and c-Myc.

**Results:** Snhg1 expression was increased in human and mouse fetal and myocardial infarction (MI) hearts, particularly in CMs. Overexpression of Snhg1 promoted CM proliferation, angiogenesis, and inhibited CM apoptosis after myocardial infarction, which further improved post-MI cardiac function. Antagonism of Snhg1 in early postnatal mice inhibited CM proliferation and impaired cardiac repair after MI. Mechanistically, Snhg1 directly bound to phosphatase and tensin homolog (PTEN) and induced PTEN degradation, activating the phosphoinositide 3-kinase (PI3K)/protein kinase B (AKT) pathway to promote CM proliferation. The c-Myc protein, one of downstream targets of PI3K/AKT signaling, functioned as a transcription factor by binding to the promoter regions of Snhg1. Perturbation of the positive feedback between Snhg1 and c-Myc by mutation of the binding sequence significantly affected Snhg1-induced CM proliferation.

**Conclusions:** Snhg1 effectively elicited CM proliferation and improved cardiac function post-MI by forming a positive feedback loop with c-Myc to sustain PI3K/Akt signaling activation, and thus may be a promising cardiac regeneration strategy in treating heart failure post-MI.

## Introduction

Cardiac regeneration represents an attractive approach for treating heart failure after myocardial infarction (MI) since it could replenish lost cardiomyocytes from the roots 1. Manipulating cell cycle entry in postnatal tissue has tremendous potential for developing therapeutic strategies for cardiac regeneration and repair after myocardial infarction 2. Many approaches have been used to control cell cycle entry in the postnatal heart, including modulation of cell cycle regulators or signaling pathways 3. Overexpression of cell cycle-related gene regulators, such as Cyclin A2, Cyclin D1, and Cyclin D2, could enhance CM cell cycle activity and improve repair following myocardial infarction (MI) 4-6. Inhibition of cell cycle suppressors, such as the transcription factor homeobox protein, also extends the time window of postnatal CM proliferation and induces CMs to re-enter the cell cycle in adult mice 7. Additionally, inhibiting the Hippo pathway allows re-entry of CMs into the cell cycle in the heart after MI and improves cardiac function in mice 8. Although manipulation of the current regeneration strategies can prolong the postnatal proliferative and regenerative windows to varying degrees, most of them likewise result in multinucleation rather than cytokinesis 2, 9. Hence, identifying new strategies that can promote cardiomyocyte cytokinesis, in addition to cell cycle entry, may be beneficial for the development of efficient therapeutic strategies for mammalian heart regeneration.

Successful cytokinesis requires efficient progression through both G1/S and G2/M phases 10. A recent study established a novel combination of Cyclin-dependent kinase 1 (CDK1), CDK4, Cyclin B1, and Cyclin D1 that efficiently induced cell division in post-mitotic cardiomyocytes 11. To control cell division, multiple positive and/or negative feedback loops are also indispensable 12. Prolonged exposure to cell cycle regulators is required for cell cycle entry and commitment to cell division 12, 13. Activation of cell cycle regulators by a positive feedback loop could sustain the activation of cell cycle re-entry and contributes to the irreversibility of cell division 14. A recent study showed that a c-Myc-driven positive feedback loop triggered epigenetic memory in embryonic stem cells to maintain their self-renewal capacity 15. However, the roles of the positive feedback loop in the induction of CM cytokinesis and heart regeneration remain unclear.

Long noncoding RNAs (lncRNAs) are a class of RNA transcripts over 200 nucleotides in length and without protein-coding potential. Previous studies by us and other groups have shown that several lncRNAs can promote CM proliferation 16, 17. The lncRNA small nucleolar RNA host gene 1 (Snhg1; Gene Bank: 23642 (human), 83673 (mouse)), which was originally identified by its oncogenic activity 18, was shown to promote cancer cell proliferation by activating the phosphoinositide 3-kinase (PI3K)/protein kinase B (AKT) signaling pathway 19, 20. PI3K/AKT signaling has also been implicated in the control of heart regeneration 21, 22. Therapeutic targeting of upstream signaling pathways that regulate cell proliferation may reduce the risk of teratogenicity 23. We predicted that Snhg1 can function upstream of PI3K/AKT signaling to control CM cytokinesis and trigger heart regeneration. Activation of PI3K/AKT signaling is known to induce the expression of CDK regulatory proteins, including c-Myc 24, and we used online tools to predict that c-Myc binds to the Snhg1 promoter. Thus, there may be a positive feedback loop between Snhg1 and c-Myc.

In this study, we demonstrated that Snhg1 and c-Myc form a positive feedback loop to sustain activation of PI3K/AKT signaling, which may induce CM cytokinesis. Once established, this Snhg1-driven self-reinforcing circuit may direct CMs to undergo cytokinesis in addition to cell cycle entry, representing a powerful approach for mammalian heart regeneration.

## Methods

### Animal model

Healthy C57BL/6J mice were purchased from the Laboratory Animal Center of Southern Medical University (Guangzhou, China). A Cre-dependent Cas9 knock-in mouse model (R26-CAG-Cas9/+) was purchased from Shanghai Model Organisms Center, Inc. α-MHC-Cre mice were provided by Dr. Kunfu Ouyang from Peking University Shenzhen Graduate School, China. MYH6-mCherry transgenic mice (MYH6*-mCherry R26-mTmG) were provided by Dr. Jinzhu Duan from Guangzhou Women and Children's Medical Center, China. All data were generated and analyzed in a blinded fashion with regard to genotype. All animal procedures conformed to the guidelines of Directive 2010/63/EU of the European Parliament on the protection of animals used for scientific purposes and the NIH Guide for the Care and Use of Laboratory Animals. Approval for this study was granted by Southern Medical University's ethics review board.

### Establishment of MI and myocardial ischemia/reperfusion (I/R) models

Mouse MI was carried out as described previously 17. P1 and P7 mice were anesthetized by cooling on an ice bed for 6 min, whereas adult male mice (8-10 weeks of age) were intraperitoneally anesthetized with 3% pentobarbital sodium (40 mg/kg) following tracheal intubation for artificial ventilation. An ALC-V8S rodent ventilator (ALCBIO, Shanghai, China) was used to supply oxygen during the surgical procedure. The chests were opened by a horizontal incision through the muscle between the fourth and fifth intercostal space. The pericardium was then removed. The left anterior descending (LAD) coronary artery was permanently ligated with a 9-0 silk suture (Ningbo Medical Needle Co., Ningbo, China). After surgery, the thoracic wall and skin were closed with a 5-0 silk suture. Myocardial ischemia was confirmed by electrocardiogram ST segment elevation using an Animal Bio Amp (ADInstruments, Bella Vista, NSW, Australia). After ligation, adenovirus (Adv) or AAV9 vectors were injected immediately into the myocardium bordering the infarct zone using an insulin syringe with a 30-gauge needle. After surgery, the skin was disinfected and the animals were revived while being kept on a thermal insulation blanket. The hearts were collected at 14 or 28 days after infarction as described below.

For the model of myocardial I/R, after occlusion of the left coronary artery for 45 min, the ligature was released for myocardium reperfusion. Successful reperfusion was confirmed by observing the return of a bright red color to the pale region in the myocardium. Sham control mice underwent the same anesthetic and surgical procedures, except the ligation of the LAD was not tied.

### Ventricular CM isolation from 1- and 7-day-old mice

1-day-old C57BL/6J mice were anesthetized with 2% isoflurane inhalation and sacrificed by cervical dislocation. The ventricles from neonatal mice were separated from the atria, cut into pieces, and digested with 0.25% trypsin (Gibco, Grand Island, NY, USA) at 4 °C overnight. Digestion was repeated twice with collagenase type II (Gibco) in phosphate-buffered saline (PBS) with bovine serum albumin (BSA) (Sigma, St. Louis, MO, USA) at 37 °C for 15 min under constant stirring. Digestion was performed at 37 ℃ in 15 min steps, and the supernatant was collected and mixed with fetal bovine serum (FBS) (Gibco) after each step. The collected supernatant was centrifuged to separate the cells, which were resuspended in Dulbecco's modified Eagle's medium/nutrient F-12 Ham (DMEM/F12) 1:1 medium (HyClone, Logan, UT, USA) supplemented with 10% FBS, 100 U/mL penicillin (Sigma), and 100 g/L streptomycin (Sigma). The collected cells were seeded onto 100-mm plastic dishes for 2 h at 37 °C in a humidified atmosphere of 5% CO_2_. The supernatant, composed mostly of CMs, was collected and pelleted. Cells were resuspended in supplemented DMEM/F12 and then counted and plated at an appropriate density.

Ventricular CMs from 7-day-old mice were isolated as described for neonatal mice, except that the digestions were performed at 37 °C in calcium- and bicarbonate-free Hanks' solution with HEPES (CBFHH) buffer containing 0.25 g/L pancreatin (Sigma), 0.125 g/L collagenase type II (Worthington Biochemical), and 10 g/L DNase II (Sigma) under constant stirring. Cells were washed thoroughly 24 h after seeding and transduction.

### CM isolation from adult mice

Adult CMs were isolated from 6- to 8-week-old mice as previously described 25. Briefly, adult mice were anesthetized with 2% isoflurane and their hearts were dissected and perfused with solution A (118 mmol/L NaCl, 4.8 mmol/L KCl, 25 mmol/L HEPES, 1.25 mmol/L MgSO_4_, 1.25 mmol/L K_2_HPO_4_, 10 mmol/L glucose, 4.95 mmol/L taurine, 9.89 mmol/L 2,3-butanedione monoxime; pH 7.35). The hearts were then adjusted on a Langendorff perfusion system and digested with digestion buffer (solution A with 0.1% BSA, 0.05 mmol/L CaCl_2_, 0.07% collagenase type II, 0.02% hyaluronidase type I). Next, ventricular tissue was removed and minced in digestion buffer. The cell suspension was filtered through a 100-μm cell strainer (BD Biosciences, Franklin Lakes, NJ, USA), and the filtrate was centrifuged for 3 min at room temperature. The cell pellet was resuspended in solution B (solution A with 1% BSA and 0.1 mmol/L CaCl_2_), and the cells were allowed to settle under gravity. This cell pellet was resuspended and seeded into DMEM/high glucose media (HyClone) containing 10% FBS, 100 U/mL penicillin, and 100 g/L streptomycin.

### Adenovirus vector and siRNA transduction *in vitro*

Adenovirus (Adv) vectors containing the green fluorescent protein (GFP) gene (Adv-GFP) for Snhg1 overexpression, si-Snhg1 and negative control (PAdM-U6-NC-GFP) were synthesized by Vigene (Shandong, China). The sequence of Snhg1 siRNA was as follows: 5ʹ-GCATTCAAAGGTTCTGTTATT-3ʹ; 5ʹ-TAACAGAACCTTTGAATGCTT-3ʹ. siRNAs for c-Myc, glycogen synthase kinase 3 beta (GSK3β) and si-NC (siN0000001-1-5) were synthesized by RiboBio (Guangzhou, China). The target sequence of c-Myc siRNA was: 5ʹ-CTATGACCTCGACTACGAC-3ʹ. The target sequence of GSK3β siRNA was: 5ʹ-GUCCUAGGAACACCAACAA-3ʹ. The phosphatase and tensin homolog (PTEN) inhibitor LY294002 were purchased from Calbiochem (Merck Eurolab, Fontenay Sous Bois, France).

Isolated mouse CMs were seeded at 70% confluence. Transduction was performed after 48 h of culture. Various multiplicity of infection (MOI) values of Adv vector were added to the cells. For siRNA transfection, 5 μL Lipofectamine 2000 (Invitrogen, Carlsbad, CA, USA) and 50 nmol/L siRNA were added to Opti-MEM medium (Gibco). The mixed solution was incubated at room temperature for 20 min and then added to the cells. After 48 h, the cells were subjected to RNA or protein isolation or immunofluorescence analysis. The transduction efficiencies of Adv vector and siRNA were determined by staining with an anti-GFP antibody (Biosynthesis, Beijing, China).

### Injection of AAV9 vectors in 7-day-old and adult mice

AAV9-GFP vectors overexpressing Snhg1 or negative control (NC, pAV-CMV-NC-GFP) were synthesized by Vigene. AAV9-mediated Snhg1 or NC were delivered by intracardiac injection into the left ventricle of 7-day-old mice at a dose of 2 × 10^10^ viral genome particles per animal using an insulin syringe with a 30-gauge needle (BD Biosciences). Adult mice received the injection at a dose of 1 × 10^11^ viral genome particles per animal. The transduction efficiency of AAV9 vector was determined by staining with an anti-GFP antibody (ab13970, Abcam). In addition, an *in vivo* imaging system (In-Vivo FX PRO; Bruker Co., Billerica, MA, USA) was used to evaluate GFP fluorescence 28 days after AAV9 vector injection (480 nm excitation). *In situ* hybridization (ISH) and real-time quantitative polymerase chain reaction (RT-qPCR) assays were used to detect Snhg1 expression after transduction.

### Statistical analysis

Data are presented as mean ± standard deviation. Statistical analyses were performed using SPSS 20.0 software (SPSS, Inc., Chicago, IL, USA). All data were subject to tests for normality. Data that do not follow a normal distribution were analyzed via a non-parametric equivalent. To statistically compare two groups, unpaired, two-tailed Student's *t*-tests were used. To compare three or more groups, one-way or two-way analysis of variance followed by the least significant difference post hoc test were used. Two-tailed *P* < 0.05 was considered to be significant. Full methods are provided in the Supplementary Information.

## Results

### Snhg1 was highly expressed in fetal and MI hearts

**Figure 1:**
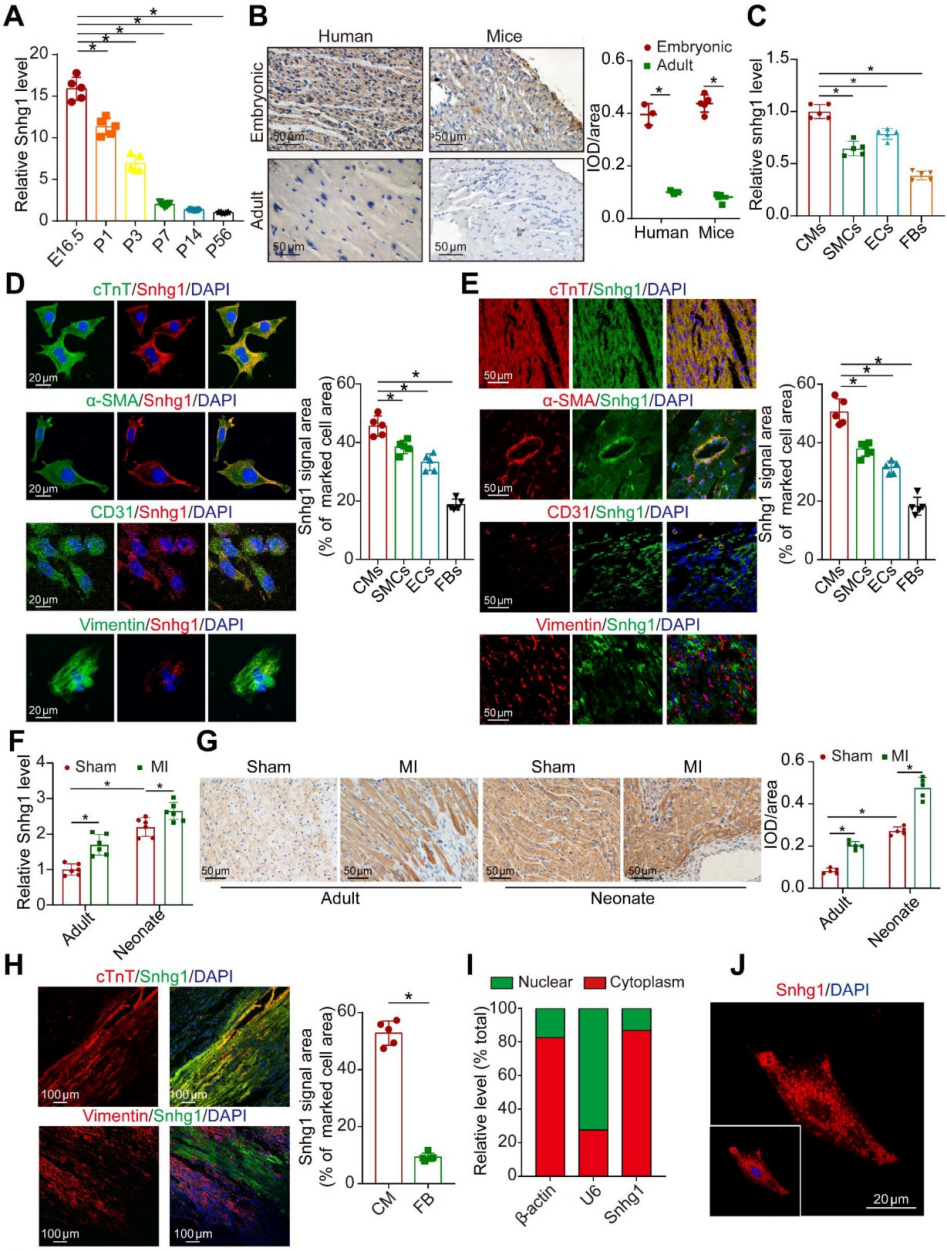
** Snhg1 was highly expressed in fetal and MI hearts. (A)** Quantification of Snhg1 expression in ventricles harvested from E16.5 to P56 mice by RT-qPCR (n = 5). **(B)** Detection of Snhg1 expression in human and mouse heart tissue by ISH (3-5 hearts). Brown dot clusters indicate Snhg1. **(C)** RT-qPCR analysis of Snhg1 in isolated neonatal cardiomyocytes(CMs), fibroblasts(FBs), endothelial cells (ECs) and smooth muscle cells (SMCs) (n = 5). **(D-E)** Co-staining of neonatal CMs, SMCs, FBs, and ECs to identify cells expressing Snhg1 by RNA-FISH *in vitro* (D) and *in vivo* (E) (n = 5). **(F)** RT-qPCR analysis of Snhg1 levels in neonatal and adult mouse hearts after MI (n = 6). **(G)** Detection of Snhg1 expression in adult and neonatal heart tissue after MI by ISH, brown dot cluster indicated Snhg1, n=5. (H) Co-staining with CM and FB markers to identify cells expressing Snhg1 in adult hearts after MI by RNA-FISH (n = 5). **(I)** RT-qPCR analysis of Snhg1 abundance in the cytoplasm and nucleus of neonatal CMs. **(J)** Detection of Snhg1expression in neonatal CMs by FISH.

Snhg1 markedly decreased during heart development from the embryonic phase (measured at embryonic day 16.5 (E16.5)) to adulthood (measured at postnatal day 56 (P56)) Snhg1(Figure 1A) and showed the most obvious change among SNHG family members (Figure S1A). Snhg1 was shown to be conserved across humans, mice, and rats (Figure S1B) and is predominantly expressed in liver, muscle, kidney, and heart tissues (Figure S1C). ISH analysis showed that Snhg1 is highly expressed in both human and murine fetal hearts (Figure 1B). We further detected Snhg1 expression in several cardiac cell types isolated from neonatal mouse hearts, and we found that Snhg1 was mainly expressed in CMs and endothelial cells (ECs) (Figure 1C). Co-staining analysis with RNA-fluorescence *in situ* hybridization (RNA-FISH) technique revealed that Snhg1 was mainly distributed in CMs (Figure 1 D-E). After MI, Snhg1 expression was upregulated in both neonatal and adult hearts, and this response is especially obvious in neonatal hearts (Figure 1F-G). We further observed that Snhg1 was mainly increased in the border zone and infarcted zone but not in the remote zone of adult hearts (Figure S1D). Co-staining analysis further revealed that the increase in Snhg1 after MI mainly occurred in CMs but not in fibroblasts (FBs) (Figure 1H). Moreover, we found that Snhg1 was mainly located in the cytoplasm of neonatal mouse CMs but not in the nucleus (Figure 1I-J). Overall, these data demonstrated that Snhg1 is highly expressed in fetal and MI hearts, particularly in CMs.

### Snhg1 promoted P7 CM proliferation* in vitro and Vivo*

**Figure 2:**
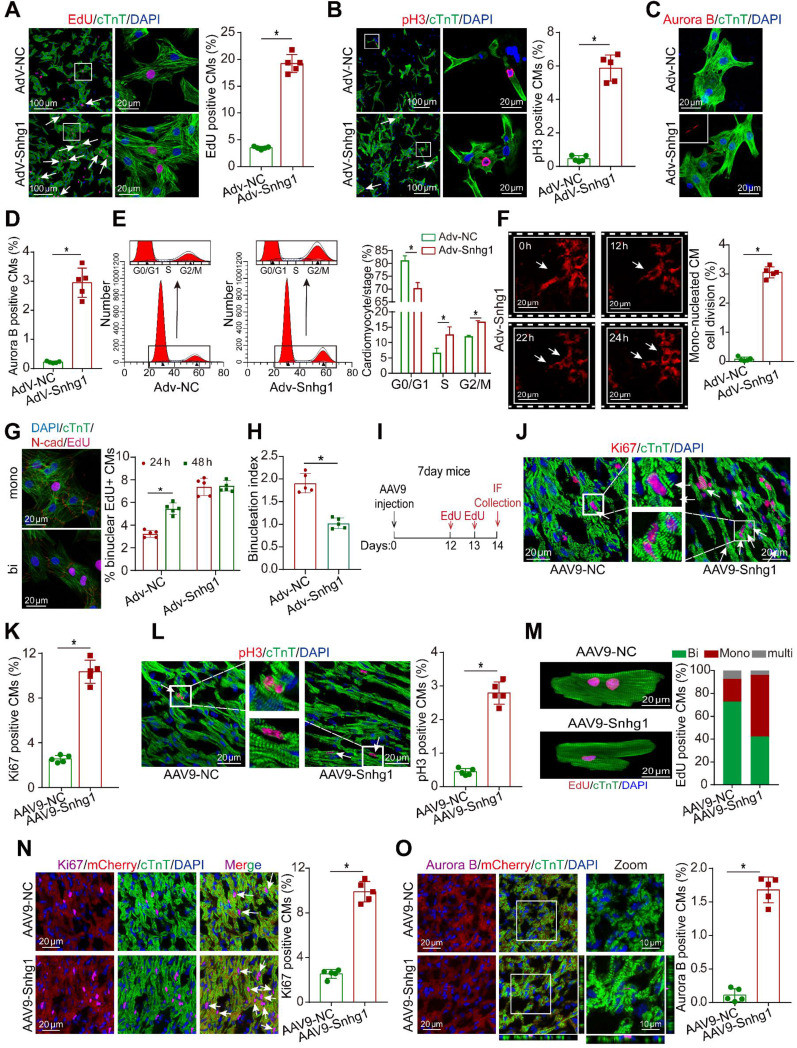
** Snhg1 overexpression promoted P7 CM proliferation. (A)** EdU staining of P7 CMs transduced with Adv-NC or Adv-Snhg1, (647 CMs from 5 mice in the Adv-NC group, 723 CMs from 5 mice in the Adv-Snhg1group). CMs were immunostained for cardiac troponin T (cTnT) and nuclei were stained with DAPI. Arrows indicate positive CMs. **(B)** Immunostaining for pH3 of P7 CMs transduced with Adv-NC or Adv-Snhg1, (467 CMs from 5 mice in the Adv-NC group, 539 CMs from 5 mice in the Ad-Snhg1 group). **(C-D)** Immunostaining for Aurora B of P7 CMs transduced with Adv-NC or Adv-Snhg1, (536 CMs from 5 mice in the Adv-NC group, 652 CMs from 5 mice in the Adv-Snhg1 group). **(E)** Detection of cell cycle alterations in P7 CMs transfected with Adv-Snhg1 or Adv-NC by flow cytometry. **(F)** Time-lapse images of Snhg1-induced cytokinesis in P7 mouse CMs isolated from MYH6-mCherry transgenic mice (427 CMs from 5 mice in the Adv-Snhg1group). Panels are representative of images recorded during cell division (see Video 1). Arrows indicate CMs undergoing cell division. **(G)** Quantification of CM binucleation in P7 CMs transduced with Adv-Snhg1 or Adv-NC. 428 CMs from 5 mice in Adv-Snhg1 group at 24 h, 476 CMs from 5 mice in Adv-NC group at 48 h, 567 CMs from 5 mice in Adv-Snhg1 group at 24 h, 536 CMs from 5 mice in Adv-NC group at 48 h. Nucleation status of EdU+ CMs was assessed by immunostaining for cardiac troponin T (cTnT), EdU and N-cadherin (N-cad). **(H)** The binucleation index, defined as the relative increase in the percentage of binuclear cells, within the EdU+ CM population from 24 to 48 h. Calculated from (G). **(I)** Schematic of the experiments in day 7 mouse hearts injected with AAV9 vectors. IF, immunofluorescence. **(J-K)** Immunostaining for Ki67 in P7 mouse hearts (n = 5). Arrows indicate positive CMs. **(L)** Immunostaining for pH3 in P7 mouse hearts (n = 5). Arrows indicate positive CMs. **(M)** EdU incorporation was detected in CM nuclei (376 CMs from 5 mice in the AAV9-NC group, 413 CMs from 5 mice in the AAV9-Snhg1 group). **(N-O)** Immunostaining for Ki67 and Aurora B in P7 MYH6-mCherry transgenic mice (n = 5). All CMs were immunostained for cTnT and nuclei were stained with DAPI. Arrows indicate positive CMs.

To investigate whether Snhg1 promoted the proliferation of P7 CMs which have limited regenerative capacity, we used an adenoviral-mediated gene transfer vector to overexpress Snhg1 in isolated P7 CMs, selected at approximately 80% purity (Figure S2A). A high transduction efficiency was observed (Figure S2B), and RT-qPCR confirmed that Snhg1 was upregulated in the Adv-Snhg1 group (Figure S2C). Overexpression of Snhg1 significantly promoted CM proliferation, as shown by staining with the DNA synthesis marker 5-ethynyl-2'-deoxyuridine (EdU) (Figure 2A) and immunostaining for the mitosis marker phosphorylated histone H3 (pH3) (Figure 2B) and the cytokinesis markers Aurora B kinase (Figure 2C-D) and cytokinetic markers Anillin (Figure S2D). Overexpression of Snhg1 promoted pH3 and Aurora B protein expression in CMs (Figure S2E). We further demonstrated that increased CM proliferation in Adv-Snhg1 group was mainly exerted by Snhg1 overexpression (Figure S2F-G). Moreover, overexpression of Snhg1 increased the CM number (Figure S2H). Flow cytometry assays revealed that Snhg1 promoted the accumulation of P7 CMs in the S and G2/M phases of the cell cycle (Figure 2E). Time-lapse imaging of primary P7 CMs labeled with the fluorescent mitochondrial dye tetramethylrhodamine ethyl ester (TMRE) showed that overexpression of Snhg1 induced mononucleated P7 CMs to undergo cell division rather than binucleation (Figure 2F, Video 1). We further applied the binucleation assay to assess the CM cytokinesis and found that Snhg1 reduced an increase in the percentage of binuclear cells within the EdU+ CMs (Figure 2G and S2I) and reduced the cardiomyocyte binucleation index (Figure 2H). The above results indicated that Snhg1 could enhance CM cytokinesis.

*In vivo,* we overexpressed Snhg1 by delivering adeno-associated virus serotype 9 (AAV9)-mediated constructs (Figure 2I). The transfection efficiency of AAV9-Snhg1 was about 80% and Snhg1 expression was significantly increased (Figure S3A-C). Overexpression of Snhg1 in P7 mice induced a marked increase in the ratio of proliferative cardiomyocytes (Figure 2J-L and Figure S3D). We used the hemocytometer method after the ventricle digestion to determinate the CM nucleation status. Overexpression of Snhg1 increased the proportion of mononucleated EdU-positive cardiomyocytes (Figure 2M), indicating that Snhg1 promoted cardiomyocyte cytokinesis. The CM-specific MYH6-mCherry transgenic mice were further used to confirm the proliferative effect exerted by Snhg1 (Figure 2N-O and Figure S3E). Collectively, the above results indicated that Snhg1 was able to induce CM proliferation both *in vitro and vivo*.

To further explore whether Snhg1 promoted CM proliferation via other cells or paracrine factors, we developed co-culture systems of CMs and FBs or ECs (Figure S4A). When CMs were co-cultured with FBs or overexpressing Snhg1-FBs, no increase in the ratio of proliferative CMs (Figure S4B-C). When CMs were co-cultured with ECs, an increase in the proliferation rate of CMs was observed (Figure S4D-E). However, overexpression of Snhg1 in ECs did not further elevate the proliferation rate of CMs (Figure S4D-E). These findings indicated that the proliferative effect exerted by Snhg1 was not through regulating the paracrine functions of FBs or ECs.

Given that Snhg1 is expressed in most cardiac cell types, we further explored whether Snhg1 affects the proliferation of FBs and macrophages. We found that overexpression or inhibition of Snhg1 had no significant effect on the proliferation of FBs (Figure S5A-B). Because only 7.2% of cultured FBs were infected when the CM Adv vector dose (10 MOI) was used, we had to increase the dose for THY1+ FBs by 10 times to reach an infection efficiency >95% (Figure S5C). We also assessed the effect of Snhg1 on macrophages and found that inhibition of Snhg1 reduced the percentage of macrophages expressing proliferation markers (Figure S5D-E). To test the role of Snhg1 in human CMs, we overexpressed Snhg1 in postmitotic 60-day-old human induced pluripotent stem cell-derived CMs (hiPS-CMs). We observed an increase in pH3+ hiPS-CMs (Figure S5F).

### Snhg1 induced adult CM proliferation

**Figure 3:**
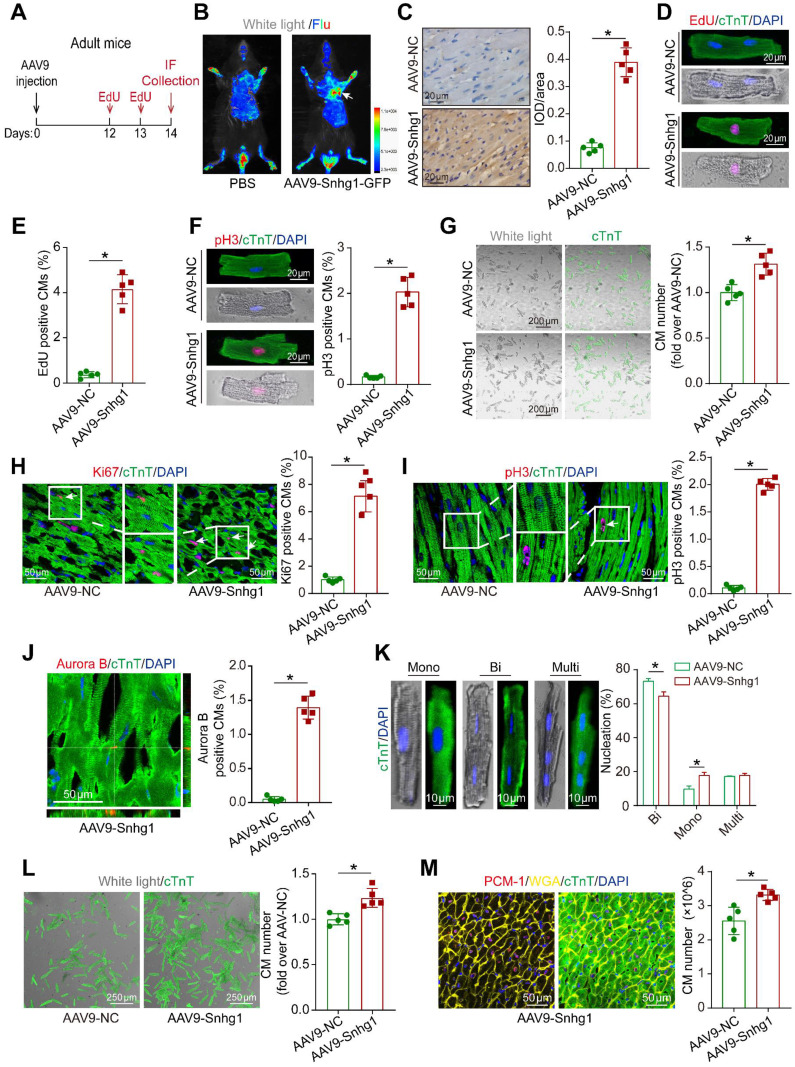
** Snhg1 promoted CM proliferation in adult mice. (A)** Schematic of the experiments in adult mouse hearts injected with AAV9. IF, immunofluorescence. **(B)** Representative *in vivo* bioluminescence and bright field images of adult mice captured on day 14 after injection of AAV9-Snhg1 virus. Arrow indicates the heart with GFP fluorescence. **(C)** ISH results confirming that Snhg1 was significantly increased in adult hearts injected with AAV9-NC or AAV9-Snhg1 (n = 5). Brown dot clusters indicate Snhg1. **(D-E)** EdU staining of CMs isolated from adult mouse hearts after transduction with AAV9-Snhg1 or AAV9-NC for 14 days (422 CMs from 5 mice in the AAV9-Snhg1 group, 359 CMs from 5 mice in theAAV9-NC group). **(F)** Immunostaining for pH3 of CMs isolated from adult mouse hearts 14 days after transduction with AAV9-Snhg1 and AAV9-NC (405 CMs from 5 mice in the AAV9-Snhg1 group, 367 CMs from 5 mice in the AAV9-NC group). **(G)** Representative bright field and cTnT immunostained images of CMs isolated from adult mouse hearts 14 days after transduction with AAV9-Snhg1 or AAV9-NC and quantification of the CM number (423 CMs from 5 mice in the AAV9-Snhg1 group, 378 CMs from 5 mice in the AAV9-NC group). **(H-J)** Immunostaining for Ki67, pH3, and Aurora B of adult mouse hearts (n=5). Arrows indicate positive CMs. **(K)** Immunostaining for cTnT of isolated CMs and quantification of the nuclei number. For nucleation analysis, ~1 × 10^3^ CMs were counted per sample (n = 5). **(L)** Quantification of isolated CM number in adult mouse hearts. **(M)** Stereological analysis revealing the number of CMs in adult mouse hearts (n = 5).

To investigate the role of Snhg1 in adult CM proliferation, we isolated CMs from adult mouse hearts injected with AAV9-Snhg1 or AAV9-NC (Figure 3A). Dual GFP fluorescence and cTnT immunostained images of CMs at high magnification confirmed that the transduction efficiency of AAV9-Snhg1 was approximately 70% and Snhg1 expression was significantly increased (Figure 3B-C and Figure S6A-D) while the transduction efficiency of AAV9-Snhg1 in other cardiac cell types was low (Figure S6E), indicating the specificity of AAV9-mediated Snhg1 overexpression in CMs. As expected, overexpression of Snhg1 in adult CMs resulted in a striking increase in cell cycle activity, and a higher CM number (Figure 3D-G). *In vivo*, overexpression of Snhg1 increased the percentage of proliferative CMs (Figure 3H-J). In addition, Snhg1 induced an increase in the percentage of mononucleated CMs. We then examined the total numbers of cardiomyocytes and nuclear state in the ventricular myocardium by enzymatic disaggregation and direct cell counting. The quantification analysis revealed an increase in the ratio of mononuclear CMs and total CM number in AAV9-Snhg1 injected hearts (Figure 3K-L). We applied stereological analysis to further confirm the increase in CM number in AAV9-Snhg1 injected hearts (Figure 3M and Figure S7A). The CM size measured by Imagepro-plus was decreased in Snhg1-overexpressing hearts (Figure S7B). However, no significant difference was observed in the heart to body weight ratios of these mice (Figure S7C). All of the above results indicated that overexpression of Snhg1 promotes adult cardiac regeneration.

### Snhg1 induced cardiac regeneration and improved functional recovery after MI

**Figure 4:**
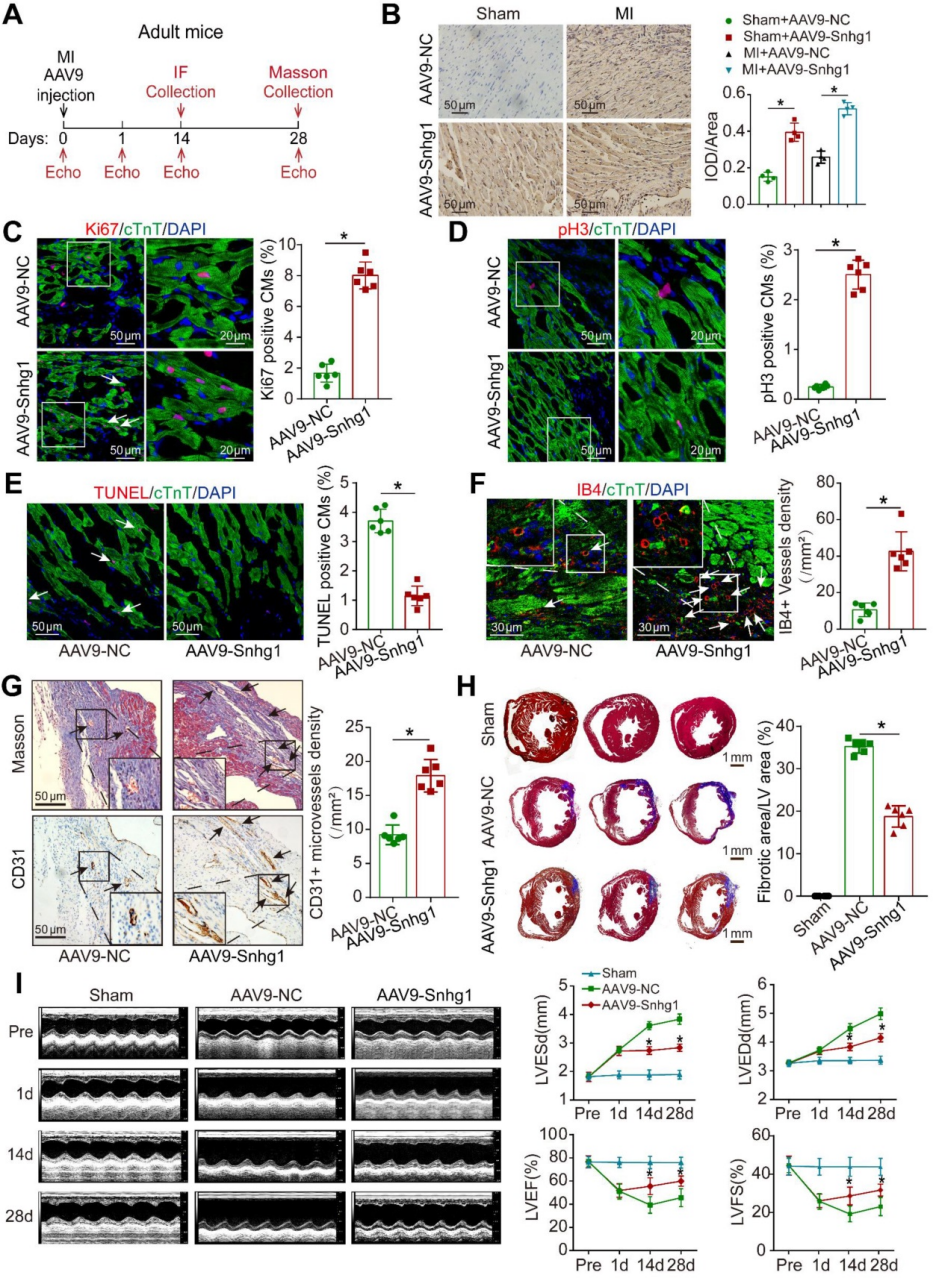
** Snhg1 improved adult cardiac function post-MI. (A)** Schematic of the MI experiments in adult mouse hearts injected with AAV9. Echo, echocardiography. IF, immunofluorescence. **(B)** Detection of Snhg1 expression in sham operated and infarcted adult mouse hearts by ISH analysis (n = 4). **(C-D)** Immunofluorescence for Ki67 and pH3 in adult mouse hearts 14 days after MI (n = 6). Arrows indicate positive CMs. **(E-F)** Immunofluorescence for TUNEL and IB4 in adult mouse hearts 14 days after MI (n = 6). Arrows indicate positive cells or vessels. **(G)** Immunohistochemistry for CD31 in adult mouse hearts 14 days after MI (n = 6). **(H)** Masson's trichrome-stained sections of adult mouse hearts 28 days after MI and quantification of infarct size (n = 6). **(I)** Echocardiography analysis of adult mouse hearts at 1, 14, and 28 days after MI (n = 10).

To determine whether Snhg1 is involved in the regulation of cardiac repair in response to ischemic injury, an MI model was induced by permanent ligation of the LAD coronary artery (Figure S8A). The peri-infarcted area was injected with AAV9 vectors expressing Snhg1 or NC (Figure 4A). The transduction efficiency of AAV9-Snhg1 in the border zone was ~80% by GFP and CM marker co-staining, which was higher than that in the remote zone and infarcted zone (Figure S8B). ISH analysis confirmed that intracardiac injection of AAV9-Snhg1 significantly increased Snhg1 expression in both sham operated and MI hearts (Figure 4B). Intramyocardial injection of AAV9-Snhg1 induced CM proliferation in the peri-infarct area (Figure 4C-D). These results were further confirmed in CM-specific MYH6-mCherry transgenic mice (Figure S8C). Overexpression of Snhg1 resulted in a decrease in myocyte apoptosis in the infarct border zone (Figure 4E) and an increase in blood vessel density in the peri-infarct area (Figure 4F-G). The CM size was significantly reduced in Snhg1-overexpressing hearts (Figure S8D). However, no significant difference was observed in the heart to body weight ratios of these mice following MI (Figure S8E). Masson's trichrome staining clearly showed that the infarct size was significantly reduced in Snhg1-overexpressing hearts (Figure 4H). As evaluated by echocardiography, Snhg1 overexpression improved cardiac function in mouse hearts at 14 and 28 days after MI (Figure 4I). The above results were confirmed in an I/R injury mouse model (Figure S9A-G). Taken together, these results indicated that Snhg1 improved cardiac repair following MI, which mainly attributed to the increase in CM proliferation and angiogenesis and reduction in CM apoptosis.

### Snhg1 depletion impaired cardiac regeneration in neonatal mice

**Figure 5:**
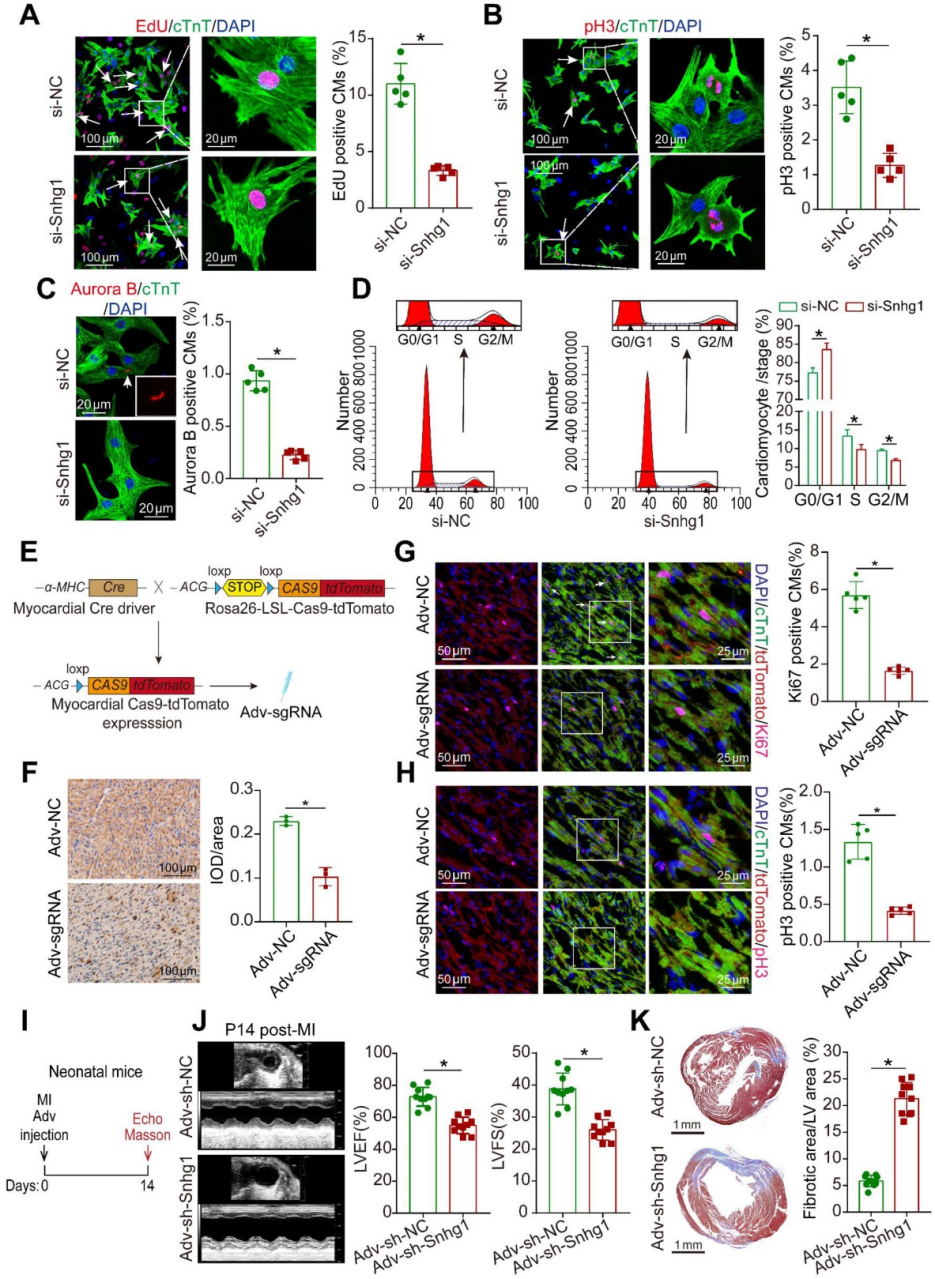
** Loss of Snhg1 impaired neonatal cardiac regeneration. (A)** Representative images and quantification of P1 CMs positive for EdU (321CMs from 5 mice in the si-Snhg1 group, 413 CMs from 5 mice in the si-NC group). Arrows indicate positive CMs. **(B)** Representative images and quantification of P1 CMs positive for pH3 (364 CMs from 5 mice in the si-Snhg1 group, 432 CMs from 5 mice in the si-NC group). Arrows indicate positive CMs. **(C)** Representative images and quantification of P1 CMs positive for Aurora B (356 CMs from 5 mice in the si-Snhg1 group, 437 CMs from 5 mice in the si-NC group). Arrows indicate positive CMs.** (D)** Detection of cell cycle alterations in P1 CMs by flow cytometry. **(E)** Schematic illustrating the procedure to construct myocardial cas9-tdTomato mouse and the delivery of Adv expressing sgRNA into heart of the myocardial cas9-tdTomato mouse. **(F)** ISH assays detecting Snhg1 expression in Cas9 mouse hearts 7 days after injection with Adv-sgRNA (Snhg1)-GFP or Adv-NC, n=3. **(G-H)** Immunofluorescence for Ki67 and pH3 in neonatal hearts 7 days after injection with Adv-sgRNA, n = 5. Arrows indicate positive CMs. **(I)** Schematic of the MI experiments in neonatal mouse hearts injected with Adv vectors. Echo, echocardiography. **(J-K)** Echocardiography results with quantification of left ventricular ejection fraction and left ventricular fractional shortening and Masson's trichrome-stained sections of neonatal mouse hearts 14 days after MI (n = 10).

We next knocked down Snhg1 in P1 mouse CMs to determine whether inhibition of Snhg1 impaired neonatal cardiac regeneration *in vitro* (Figure S10A). Depletion of Snhg1 decreased the percentage of proliferative CMs, and induced CMs were accumulated at the G0/G1 phase of the cell cycle (Figure 5A-D). The pH3 and Aurora B protein expressions were also decreased (Figure S10B). To further assess the *in vivo* effect of Snhg1 downregulation, we delivered Adv-sh-Snhg1 to neonatal mice and detected the proliferation of cardiomyocytes (Figure S11A). Snhg1 was decreased in neonatal mouse hearts after intracardiac injection of Adv-sh-Snhg1 for 5-7 days (Figure S11B-C). Inhibition of Snhg1 resulted in a decrease in CM proliferation (Figure S11D-G). We further constructed CM-specific Snhg1 knockout mice using CRISPR/Cas9 technology (Figure 5E and Figure S12A). cTnT/Cas9 co-staining confirmed that Cas9 was expressed in CMs (Figure S12B), indicating that the CM-specific Cas9 model was successfully constructed. The transduction efficiency of Adv-sgRNA in the heart tissue was ~70% (Figure S12C).

Whole Genome Resequencing (WGS) was applied to determine knockout efficiency. Analysis of base composition revealed that the DNA-seq data was balanced along reads (Figure S12D) and the distribution of qualities confirmed that the sequencing quality of this lane was high (Figure S12E). The knockout efficiency of sgRNAs were confirmed by analysis of sequencing data using the Burrows-Wheeler Alignment (BWA) tool (Figure S12F). RT-qPCR and ISH assays also confirmed the efficient deletion of Snhg1 expression in Cas9-tdTomato mouse hearts (Figure S12G and Figure 5F). Knock out of Snhg1 specifically in CMs was observed to markedly decrease the proportion of proliferative CMs (Figure 5G-H). Then we determinate whether knockdown of Snhg1 affected cardiac repair after MI in neonatal mice (Figure 5I). ISH assays confirmed the efficient deletion of Snhg1 expression in both sham operated and MI neonatal hearts by Adv-sh-Snhg1 (Figure S12H). At 14 days post-MI, systolic function was significantly impaired (Figure 5J) and the fibrosis area was greater in the Adv-sh-Snhg1 group (Figure 5K). Collectively, these results suggested that downregulation of Snhg1 impaired neonatal cardiac regeneration.

### Snhg1 regulated cardiac regeneration through the PTEN/PI3K-AKT pathway

**Figure 6:**
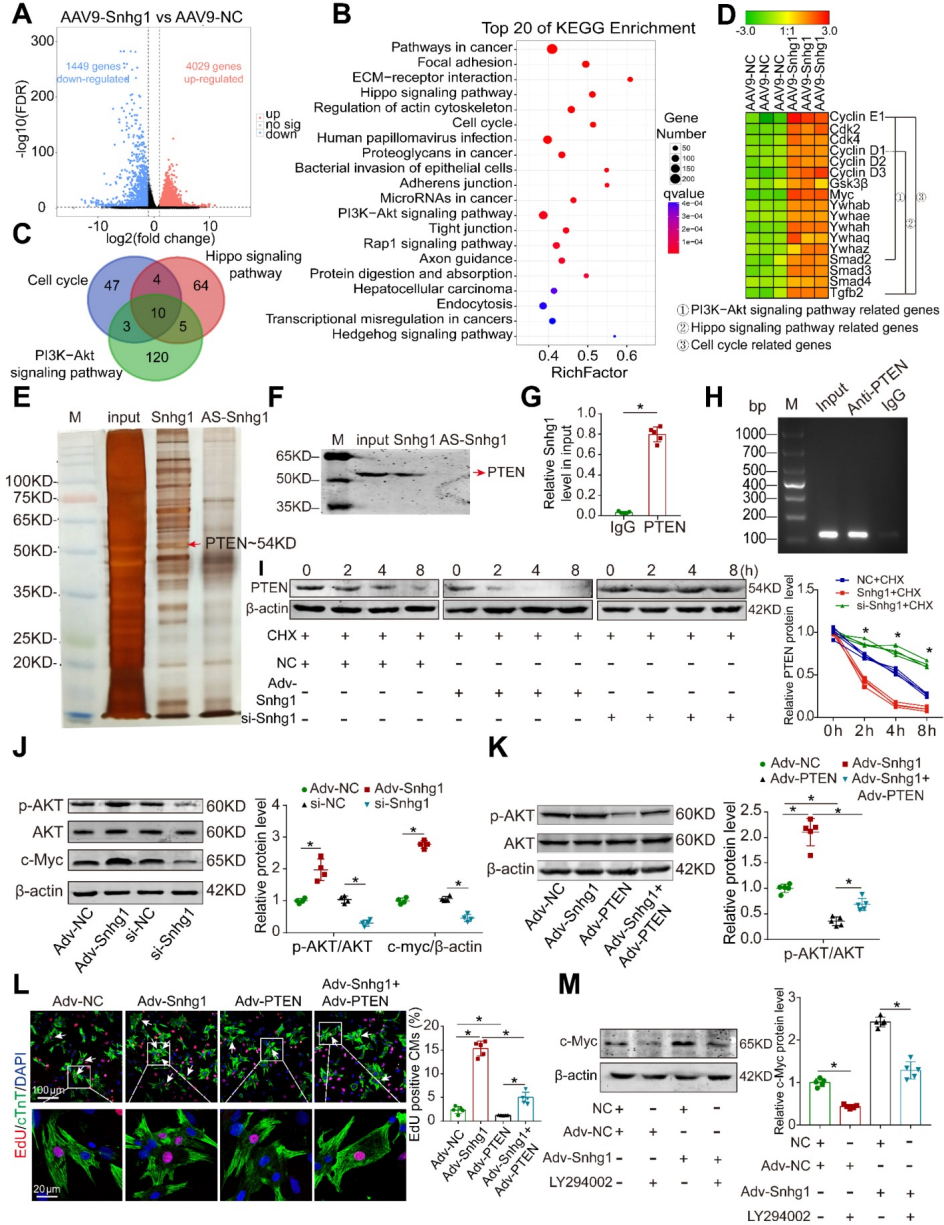
** Snhg1 regulated CM proliferation via the PTEN/AKT/c-Myc pathway. (A)** Volcano plot displaying the differentially expressed genes in P7 hearts. Red dots represent up-regulated expressed genes and blue dots represent downregulated expressed genes. **(B)** KEGG pathway enrichment scatter plot of the top 20 upregulated genes. x-axis indicates the enrichment factor, y-axis specifies the KEGG pathway. **(C)** Venn diagram showing the number of enriched KEGG pathway genes overlapping the cell cycle, PI3K-AKT, and Hippo pathways. **(D)** Representative gene modules of overlapping genes. **(E)** Silver-stained SDS-PAGE gel of proteins immunoprecipitated by an RNA pull-down assay of Snhg1 or its antisense RNA (AS-Snhg1). Arrow indicates the identified PTEN protein. **(F)** PTEN protein assayed by Western blotting. **(G-H)** RIP assay performed using an antibody against PTEN or negative IgG (n = 5). Purified RNA was used for RT-qPCR analysis, and enrichment of Snhg1 was normalized to the input. **(I)** PTEN protein levels measured by Western blotting in isolated CMs (n = 4). CHX was used to block protein synthesis. **(J)** Western blotting analysis of p-AKT, AKT, and c-Myc protein in isolated CMs with Snhg1 overexpression or depletion (n = 4). **(K)** Western blotting analysis of p-AKT and AKT protein levels. **(L)** EdU staining of isolated CMs (307 CMs from 5 mice in the Adv-NC group, 437 CMs from 5 mice in the Adv-Snhg1 group, 328 CMs from 5 mice in the Adv-PTEN group, 375 CMs from 5 mice in the Adv-Snhg1+ Adv-PTEN group). Arrows indicate positive CMs. **(M)** Western blotting analysis of c-Myc protein levels in isolated CMs.

To explore how Snhg1 mediates cardiac regeneration, we performed next-generation RNA sequencing in P7 mice overexpressing Snhg1. The transcriptome analysis identified 4029 genes that were significantly upregulated and 1449 genes that were significantly downregulated (Figure 6A and S13A). The upregulated genes are associated with cell development or differentiation (Figure S13B). The top enriched pathways of differentially upregulated genes include cell cycle, PI3K-AKT, and Hippo signaling pathways (Figure 6B), which play vital roles in CM regeneration. The differentially expressed genes enriched in the cell cycle, PI3K-AKT, and Hippo signaling pathways overlapped (Figure 6C-D, Figure S13C). These findings suggest that Snhg1 plays a prominent role in the mediation of CM proliferation.

We further performed RNA pull-down assays in isolated P7 CMs to identify the protein that interacts with Snhg1(Figure 6E). Mass spectrometry identified PTEN as the possible protein interacting with Snhg1 (Figure S14A). Western blotting and RNA immunoprecipitation (RIP) assays verified the interaction between Snhg1 and PTEN (Figure 6F-H). Overexpression of Snhg1 decreased the protein levels of PTEN, and knockdown of Snhg1 showed the opposite effect on PTEN protein (Figure S14B). Using the protein synthesis inhibitor cycloheximide (CHX), we found that Snhg1 decreased PTEN via altering its stability (Figure 6I). We further investigated whether Snhg1 regulates the PI3K-AKT pathway via PTEN. Overexpression of Snhg1 increased phosphorylated AKT (p-AKT) and c-Myc protein levels, while depletion of Snhg1 showed the opposite effects (Figure 6J). Rescue of PTEN counteracted the increase in p-AKT protein levels and the proliferative effect by Snhg1 overexpression (Figure 6K-L and S14C-E). Inhibition of AKT phosphorylation using LY294002 abrogated the elevated protein expression of c-Myc induced by Snhg1 (Figure 6M). Next, we explored how AKT regulates c-Myc. Depletion of Snhg1 or inhibition of AKT phosphorylation decreased both phosphorylation of GSK3β and c-Myc protein levels (Figure S14F). Also, the decrease in c-Myc protein levels caused by LY294002 was partly restored by inhibition of GSK3β (Figure S14G). Thus, Snhg1 induced AKT phosphorylation by decreasing the stability of PTEN, leading to inhibition of GSK3β, thereby preventing proteasome-mediated proteolysis of c-Myc. All of the above results demonstrated that Snhg1 increased PTEN degradation, leading to increased Akt and PI3K phosphorylation and subsequent activation of PI3K/Akt signaling.

We further explored how snhg1 regulates angiogenesis. Overexpression of Snhg1 increased the protein levels of vascular endothelial growth factor A (VEGFA) and decreased the protein levels of phosphorylated GSK3β (p-GSK3β), while depletion of Snhg1 showed the opposite effects (Figure S15A). Inhibition of AKT phosphorylation by LY294002 abrogated the changes in VEGFA and p-GSK3β protein expressions induced by Snhg1 (Figure S15B). These data indicate that Snhg1 regulates angiogenesis by promoting VEGFA expression and GSK3β phosphorylation.

### c-Myc/Snhg1 formed a positive feedback loop

**Figure 7:**
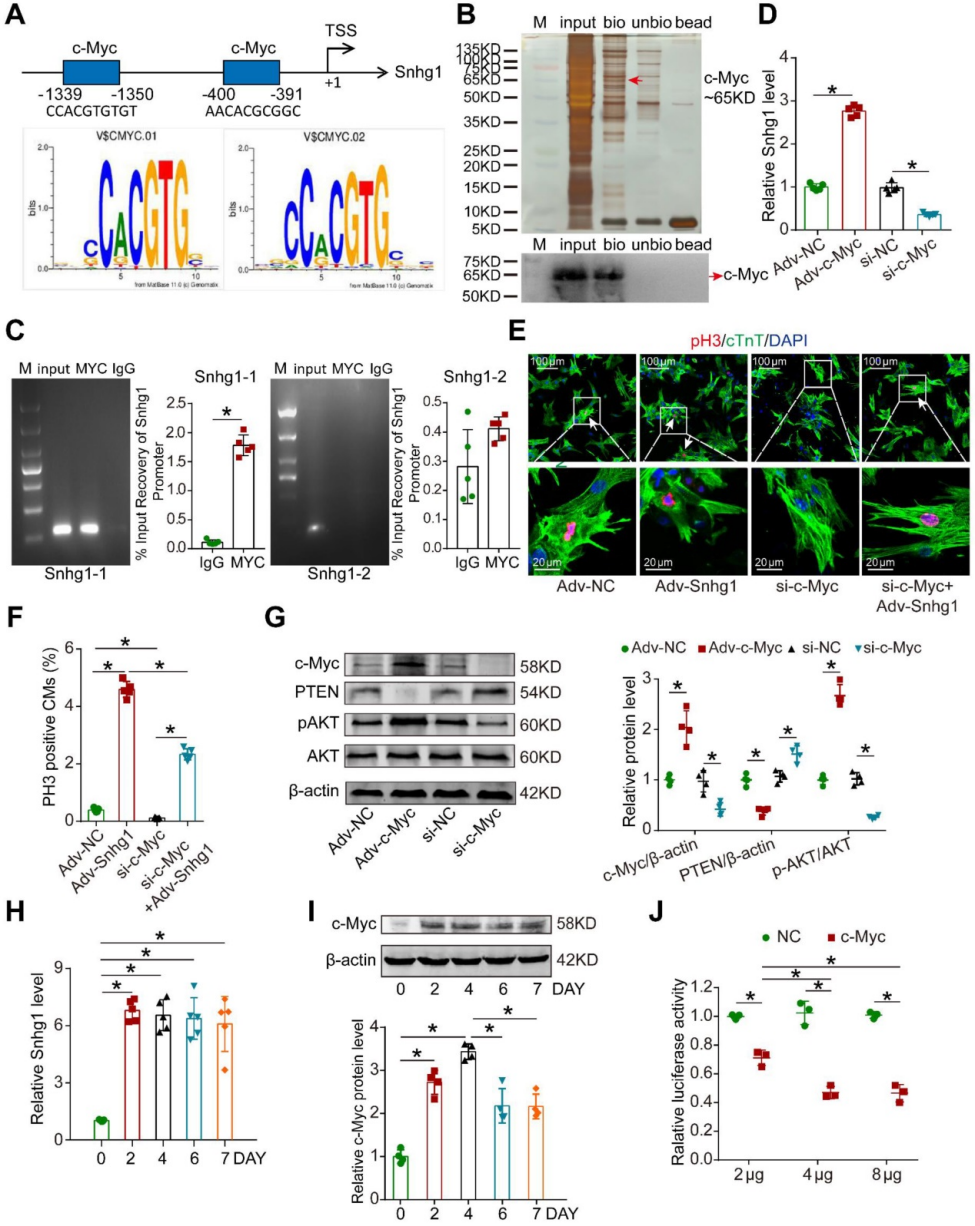
** c-Myc upregulated Snhg1 expression by binding to its promoter region. (A)** The two predicted c-Myc binding regions and sequences in the promoter region of Snhg1. **(B)** Silver-stained SDS-PAGE gel of proteins immunoprecipitated by a DNA pull-down assay of a biotinylated or unbiotinylated probe targeting Snhg1. Arrow indicates region of the gel excised for MS determination. c-Myc protein was assayed by Western blotting. **(C)** Image of an agarose gel for ChIP-qPCR using anti-c-Myc or anti-IgG antibodies to access binding between Snhg1 and c-Myc (n = 5). Purified RNA was used for RT-qPCR, and enrichment of Snhg1 was normalized to the input. **(D)** RT-qPCR analysis of Snhg1 levels in isolated P7 CMs of various groups (n = 5). **(E-F)** Immunofluorescence for pH3 of P7 CMs transfected with Adv-NC, Adv-Snhg1, si-c-Myc, or si-c-Myc + Adv-Snhg1 (327 CMs from 5 mice in the Adv-NC group, 439 CMs from 5 mice in the Adv-Snhg1group, 309 CMs from 5 mice in the si-c-Myc group, 352 CMs from 5 mice in the si-c-Myc + Adv-Snhg1 group). Arrows indicate positive CMs. **(G)** Western blotting analysis of c-Myc, PTEN, p-AKT, and AKT protein levels in isolated CMs overexpressing or depleted of c-Myc (n = 4). **(H)** RT-qPCR analysis of Snhg1 levels in isolated P7 CMs transfected with Adv-Snhg1 after 0, 2, 4, 6, and 7 days (n = 5).** (I)** Western blotting analysis of c-Myc protein levels in isolated P7 CMs transfected with Adv-Snhg1 after 0, 2, 4, 6, and 7 days (n = 4). **(J)** Luciferase assay of P7 CMs transfected with the c-Myc promoter (PGL3-c-Myc plasmid) and various concentrations of c-Myc protein (n = 3). The background luciferase activity (empty vector) was subtracted from all data.

We next identified the upstream regulator of Snhg1. Using the Jaspar database, we predicted that c-Myc has two potential binding sites in the Snhg1 promoter (Figure 7A). We further found that c-Myc functioned as a transcription factor by binding to the promoter regions of Snhg1 and upregulating the Snhg1 expression level (Figure 7B-D). Moreover, inhibition of c-Myc counteracted the proliferative effect of Snhg1 overexpression (Figure 7E-F). Overexpression of c-Myc increased p-AKT expression and decreased PTEN expression in CMs, while suppression of c-Myc showed the opposite effects (Figure 7G).

Since c-Myc was one downstream target of PI3K/Akt signaling, the consequent potentiation of the PTEN/PI3K/Akt signaling increased c-Myc levels, which in turn activated Snhg1. These data revealed a positive feedback loop between c-Myc and Snhg1.

We further explored whether this positive feedback loop is self-limiting over time. We found that overexpression of Snhg1 increased the level of Snhg1 starting from day 2, which remained stable for at least 7 days, while the levels of its downstream protein c-Myc declined on day 6 and became stable thereafter (Figure 7H-I). Luciferase assays revealed that as the level of c-Myc protein increased the inhibition of c-Myc promoter activity increased and gradually reached steady state (Figure 7J), indicating that c-Myc drives an autoregulatory feedback loop.

### Perturbation of the positive feedback inhibited CM proliferation exerted by Snhg1

**Figure 8:**
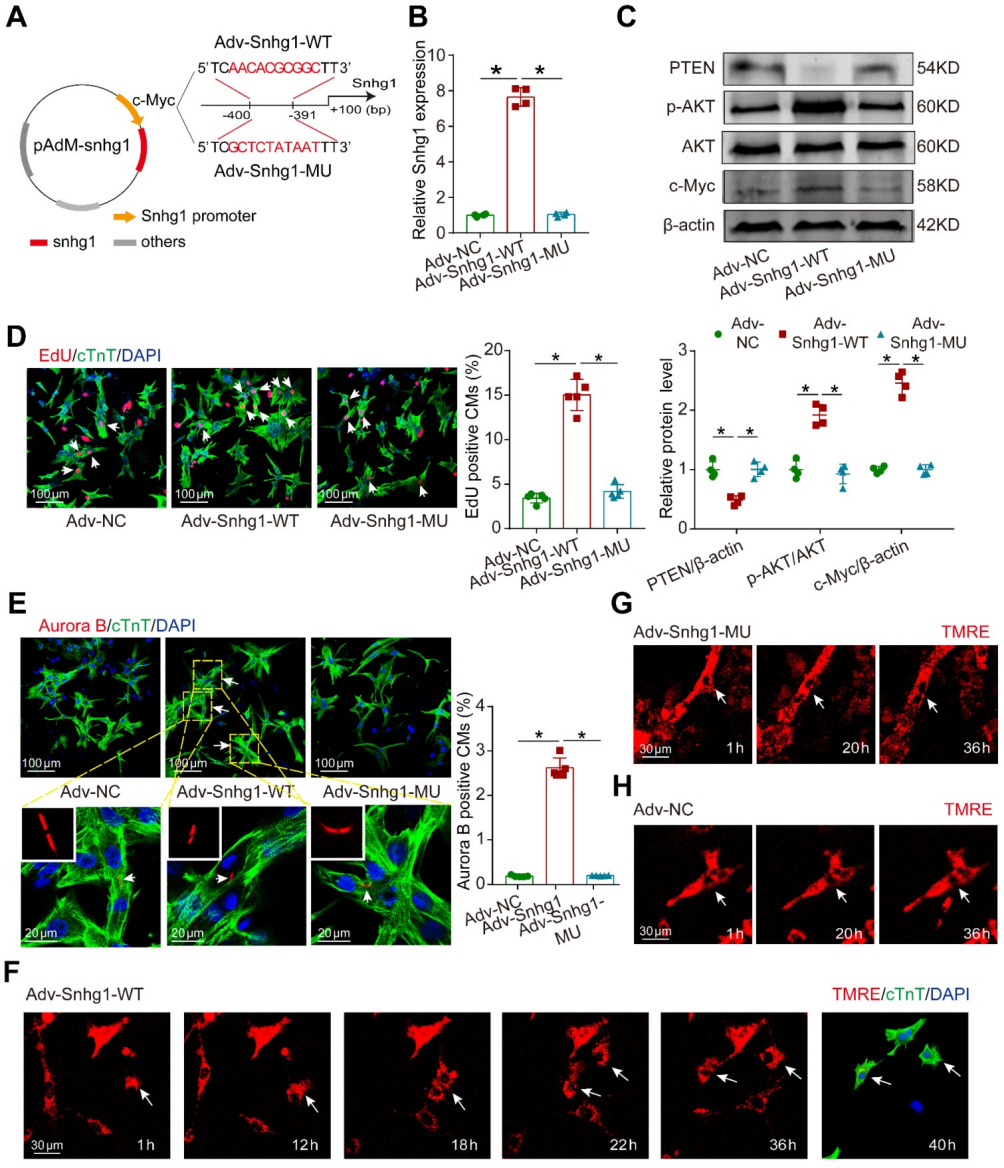
** Mutant Snhg1 inhibited Snhg1-induced CM proliferation. (A)** Construction of the overexpression vectors Adv-Snhg1-WT and Adv-Snhg1-MU. **(B-C)** RT-qRCR analysis of Snhg1 levels and Western blotting analysis of PTEN, PI3K, p-AKT, AKT, and c-Myc protein levels in isolated CMs (n = 4). **(D)** EdU staining of P7 CMs (427 CMs from 5 mice in the Adv-Snhg1-WT group, 387 CMs from 5 mice in the Adv-Snhg1-MU group). Arrows indicate positive CMs. **(E)** Aurora B immunofluorescence staining of P7 CMs (326 CMs from 5 mice in the Adv-NC group, 406 CMs from 5 mice in the Adv-Snhg1-WT group, 354 CMs from 5 mice in the Adv-Snhg1-MU group). Arrows indicate positive CMs. **(F-H)** Representative images from time-lapse videos of P7 CMs transduced with Adv-Snhg1-WT (F), Adv-Snhg1-MU (G), or Adv-NC (H). Representative images from time-lapse videos of P7 CMs transfected with Adv-Snhg1-WT (F), Adv-Snhg1-MU (G), or Adv-NC (H). CMs grown for 36 h immunostained for cTnT and stained with DAPI.

In order to demonstrate that the positive feedback between Snhg1 and c-Myc is key for Snhg1 to induce CM proliferation, we constructed Snhg1 overexpression vectors using the wildtype (Snhg1-WT) or a mutant (Snhg1-MU) c-Myc-binding sequence as the promoter (Figure 8A). After the c-Myc-binding sequence was mutated, overexpression of Snhg1 (Snhg1-MU) no longer increased snhg1 levels (Figure 8B). Luciferase assays further revealed that overexpression of c-Myc could not increase the Snhg1 promoter activity when the c-Myc-binding sequence was mutated (Figure S16A-B), indicating that c-Myc binding to the Snhg1 promoter is key for Snhg1 transcription rather than the level (shortage) of c-Myc expression (Figure S16C). In addition, overexpression of Snhg1 did not affect the expressions of PTEN, p-AKT, or c-Myc (Figure 8C) or CM proliferation (Figure 8D-E) when the c-Myc-binding sequence was mutated. To directly visualize CM division, we conducted time-lapse imaging of P7 CMs labeled with the fluorescent mitochondrial dye TMRE. We observed that Snhg1 overexpressing CMs underwent cytokinesis (Figure 8F, Video 2), while karyokinesis of CMs was observed when the c-Myc-binding sequence was mutated (Figure 8G, Video 3) and no cell division was observed in the NC group (Figure 8H, Video 4). These results indicated that the positive feedback between Snhg1 and c-Myc is key for Snhg1 to induce CM proliferation.

## Discussion

In this study, we determined the central role of Snhg1 in regulating cardiac regeneration and repair after MI. Overexpression of Snhg1 increased CM proliferation and improved post-MI cardiac function in both P7 and adult mice. In contrast, antagonism of Snhg1 impaired neonatal cardiac regeneration. Mechanistically, Snhg1 formed a positive feed-back loop with c-Myc to drive a self-reinforcing circuit that sustained activation of the PTEN/PI3K/AKT pathway, resulting in continuous cell cycle re-entry. Altogether, we shed light on the fact that Snhg1 is capable of stimulating cardiac regeneration, which represent as a promising therapeutic target for heart failure.

Our study provided important insights into the role of Snhg1 in regulating postnatal cardiac regeneration. Increasing evidence has shown that the adult mammalian heart can undergo some self-renewal at a low level 26. However, few or no CMs undergo cytokinesis to form new daughter cells 27. Attempts to control CM cytokinesis are of great interest to the fields of cardiac development and regeneration. Intramyocardial injection of Adv vectors carrying complementary DNA encoding myocardial Cyclin A2 was previously shown to induce cytokinesis of adult CMs. However, directly regulating the cell cycle may increase the risk of teratogenicity. Therapeutic targeting of upstream signaling pathways may prevent this effect. In the current study, we found that Snhg1 functioned upstream to sustain activation of the PTEN/PI3K-AKT pathway, resulting in CM proliferation. We utilized rigorous strategies to evaluate proliferative effects, including nucleotide Analogues (EdU) detection, cell cycle markers (Ki67) analysis and classical cell mitosis and cytokinesis marker (pH3 and Aurora B kinase) analysis. The proliferative effects exerted by Snhg1 were confirmed by using CM-specific MYH6-mCherry transgenic mice. Because relying entirely on proliferation or mitosis markers may misrepresent actual cytokinesis, we performed an *in vitro* cytokinesis assessment to confirm the incidence of cytokinesis in real time through the use of time-lapse fluorescence microscopy. In addition, we applied the binucleation assay to assess the cardiomyocyte cytokinesis which was proven to be reliable readout of cardiomyocyte cytokinesis 28. Furthermore, we calculated CM number using and hemocytometer method after the ventricle digestion and design-based stereology analysis 29. Our results demonstrated the ability of Snhg1 to promote CM cytokinesis in addition to cell cycle re-entry.

We further investigated the mechanisms through which Snhg1 promoted cardiomyocyte proliferation and found that Snhg1 bound to PTEN and increases its degradation, leading to increased AKT phosphorylation and subsequent activation of PI3K/AKT signaling. A very recent study implicated that the loss of PTEN directly promotes cardiomyocyte proliferation to enhance myocardial repair in response to MI 30. In this study, we found that Snhg1 bound to PTEN and increased its degradation. The degradation of PTEN is mainly regulated by phosphorylation, acetylation and ubiquitination. Of note, ubiquitination of PTEN draws intensive attention recently as it plays a major role in PTEN stability as well as nuclear localization 31. Several studies reported that ubiquitination of PTEN via regulating NEDD4-1-mediated poly-ubiquitination 32. LncRNAs or circRNA often interact with RNA-binding proteins to fulfill their regulatory functions and our previous study reported that circNfix interacted with Ybx1 and promoted Ybx1 degradation through ubiquitination-proteasome pathways 25. We suspected Snhg1 is very likely functions as a novel binding partner of NEDD4-1 to activate its E3 ligase activity and thus regulating NEDD4-1-mediated PTEN poly-ubiquitination and cell proliferation. The PTEN degradation leads to activation of PI3K-AKT signaling, which is thought to promote proliferation and increase cell survival, including mammalian CM proliferation and heart regeneration^23^. PI3K/AKT signaling not only affects the G0/G1 transition but also activates this signaling in late G1, an event required for S phase entry 24. Our data suggested that Snhg1 is a key upstream regulator of PTEN that modulates AKT phosphorylation by activating the PI3K/AKT pathway which controlled cell division. In addition to promoting CM proliferation, we also demonstrated that Snhg1 promotes angiogenesis by activating the PI3K/AKT pathway.

Interestingly, the c-Myc, one downstream target of PI3K/Akt signaling, functioned as a transcription factor by binding to the promoter regions of Snhg1 to form a positive feedback loop. Positive feedback plays an integral role in cellular differentiation, development, and cancer progressionn^14^. Additionally, positive feedback links many regulatory genes (such as c-Myc) into circuits, which is a key mechanism ensuring that that a cell is fully committed to cell division and that the events occur in order 12. If the positive feedback is perturbed, mitosis can become considerably longer and variable, causing cells to die during mitosis or sooner after without reaching a second round of cell division 33. In this study, we identified the critical interplay between Snhg1 and PI3K/Akt signaling, which drives the onset of a positive feedback loop through transcriptional activation of c-Myc genes. In addition, we perturbed this positive feedback by mutation of c-Myc-binding sequence of promoter of Snhg1 to demonstrate the positive feedback between c-Myc and Snhg1 was the key is key for Snhg1-induced CM proliferation. Since ongoing cellular division triggered by the positive feedback loop may increase the risk of tumorigenesis, we further found that the effect of Snhg1 on CM proliferation is self-limiting as time progresses. Our findings potentiated the ability of Snhg1 to promote CM proliferation.

Although our data showed that Snhg1 is promising for cardiac regeneration, there are some potential limitations. Positive feedback loops tend to lead to instability, and a lack of control may cause ongoing cellular division. Because excessive or unwanted cell division may increase the risk of tumorigenesis, delivery of Snhg1 must be carefully controlled. Transient *in vivo* delivery and organ-specific delivery systems may reduce the risks associated with tumorigenesis. Although we did not observe any cardiac tumors in mice treated with Snhg1, the potential for ectopic proliferation requires careful and specific delivery in future clinical development. Another risk associated with viral delivery systems is activation of an immune response. The immune system may block the virus from delivering the gene to the heart, impacting the effectiveness of Snhg1. Given the pleiotropic effects of lncRNAs, effective translation of Snhg1-based treatments into successful clinical therapies may require a deeper understanding of the underlying molecular and cellular mechanisms driving Snhg1-induced CM proliferation. Moreover, these results must be further validated in large animal models for cardiac regeneration post-MI or other ischemic injury.

## Conclusion

In summary, our data showed that Snhg1 formed a positive feedback loop with c-Myc to sustain activation of PI3K/AKT signaling, which effectively elicited CM cytokinesis and improved function post-MI. These findings suggested that Snhg1 might represent as a powerful regenerative approach in treating heart failure.

## Supplementary Material

Supplementary figures and tables.

Supplementary Video 1.

Supplementary Video 2.

Supplementary Video 3.

Supplementary Video 4.

## Abbreviations

AAV9: adeno-associated virus 9
Adv: adenovirus
AKT: protein kinase B
BSA: bovine serum albumin
BWA: Burrows-Wheeler Aligner
CMs: cardiomyocytes
CDK1: Cyclin-dependent kinase 1
ChIP: chromatin-immunoprecipitation
CHX: cycloheximide
cTnT: cardiac troponin T
DMEM: Dulbecco's modified Eagle medium
E16.5: embryonic day 16.5
ECs: endothelial cells
EdU: 5-ethynyl-2'-deoxyuridine
FBs: Fibroblasts
FBS: fetal bovine serum
FISH: fluorescent *in situ* hybridization
hiPS-CMs: human induced pluripotent stem cell-derived CMs
GFP: green fluorescent protein
GSK3β: glycogen synthase kinase 3 beta
HCl: hydrochloric acid
I/R: ischemia/reperfusion
ISH: *In situ* hybridization
LAD: ligating the left anterior descending
lncRNAs: long non-coding RNAs
LVEDd: diastolic left ventricular dimensions
LVEDs: left ventricular dimensions
LVEF: left ventricular ejection fraction
LVFS: left ventricular fractional shortening
LSD: least significant difference
MI: myocardial infarction
MS: mass spectrometry
N-cad: N-cadherin
P1: postnatal day 1
p-AKT: phosphorylated AKT
p-GSK3β: phosphorylated GSK3β
pH3: phospho-histone H3
PI3K: phosphoinositide 3-kinase
PTEN: phosphatase and tensin homolog
RIP: RNA-binding immunoprecipitation
RT-qPCR: quantitative RT-PCR
SDS-PAGE: sodium dodecylsulfate polyacrylamide gel electrophoresis
siRNA: small interfering RNA
Snhg1: Small nucleolar RN A host gene 1
SMCs: smooth muscle cells
TMRE: tetramethylrhodamine ethyl ester
TSS: transcription start site
VEGFA: Vascular endothelial growth factor A
WGA: wheat germ agglutinin
