# Supplementary material for: LncRNA Snhg1-driven self-reinforcing regulatory network promoted cardiac regeneration and repair after myocardial infarction
Source: Theranostics. 2021 Sep 13;11(19):9397–414. doi: 10.7150/thno.57037 (PMC8490501; doi:10.7150/thno.57037)
Supplement: Supplementary file 1 — Supplementary figures and tables. [file thnov11p9397s1.pdf]

LncRNA Snhg1-driven self-reinforcing regulatory network promotes cardiomyocyte regeneration and repair after myocardial infarction

Mengsha Li[1,2,3,*], Hao zheng[1,2,3,*], Yuan Han[1,2,3,*], Yijin Chen[1,2,3] , Bing Li[1,4], Guojun Chen[1,2,3], Xiaoqiang Chen[1,2,3], Senlin Huang[1,2,3], Xiang He[1,2,3],Guoquan Wei[1,2,3], Tong Xu[1,2,3], Xiaofei Feng[1,2,3], Wangjun Liao[5], Yulin Liao[1,2,3], Yanmei Chen[1, 1,2,3, †], Jianping Bin[1,2,3†]

[1]Department of Cardiology and National Key Lab for Organ Failure Research, Nanfang Hospital, Southern Medical University, Guangzhou, 510515, China

[2]Bioland Laboratory (Guangzhou Regenerative Medicine and Health Guangdong Laboratory), Guangzhou 510005, China.

[3]Guangdong Provincial Key Laboratory of Shock and Microcirculation, Nanfang Hospital, Southern Medical University, Guangzhou 510515, China.

[4]School of Medicine, Guizhou University, Guiyang, Guizhou, 550025, China.

[5]Department of Oncology, Nanfang Hospital, Southern Medical University, Guangzhou, 510515, China.

*These authors contributed equally.

[†]**Corresponding author:** Jianping Bin (jianpingbin@126.com) and Yanmei Chen (yanmei0812@126.com).

Running title: LncRNA Snhg1 promotes cardiac regeneration

Detailed Methods

Human tissue samples

Human left ventricular tissue samples were obtained as described previously.[1] Briefly, adult myocardial tissue samples were obtained by endomyocardial biopsy from patients suffering from myocardial deposition disease based on arrhythmia and echocardiographic changes. The pathological findings showed no evidence of myocardial disease or functional abnormalities. Embryonic human myocardial samples were obtained after elective termination of pregnancy for nonmedical reasons. The present study conformed to the principles of the Declaration of Helsinki. The study protocol was approved by the Nanfang Hospital ethics committee, and written informed consent was obtained from all subjects.

Human iPSC-CM cultures

CMs derived from iPSCs were purchased from Cellapy (Beijing, China) and were cultured according to the manufacturer's instructions. The cells were allowed to adhere for 48 h before maintenance medium exchange and fresh medium was replaced every other day and then for transduction experiments.

Isolation and culture of neonatal FBs

FBs were isolated from mice as previously described.[2] Excised hearts were rinsed in cold Hank's balanced salt solution (HBSS), minced, and digested with type II collagenase and

pancreatin at 37 °C for 15 min. The first digestion was discarded. A second digestion was performed, and the collagenase medium containing FBs was collected, centrifuged, and resuspended in DMEM with 10% FBS, 100 U/mL penicillin, and 100 g/L streptomycin. Digestion was repeated 5-6 times until the digestion fluid became clear. Cells were plated in 60-mm dishes and allowed to attach for 60 min, and the media was then changed to remove CMs and endothelial cells. Isolated FBs were washed twice with PBS and cultured for transduction experiments.[1]

Myocardial Cas9 knockin transgenic mouse model

The Cre-dependent Cas9 knockin mouse model was obtained from Shanghai Model rganisms Center, Inc. This model was generated by homologous recombination in JM8A3 embryonic stem (ES) cells and implanted in C57BL/6J blastocysts as standard procedures. Briefly, the targeting vector was designed to contain a ubiquitously expressed CAG promoter, a loxP-flanked PGK-Neo-polyA sequence followed by a Cas9 protein inserted into intron 1 of the Rosa26 locus. The construct was linearized and electroporated into JM8A3 embryonic stem cells. Targeted single-ES cell colonies were screened by PCR with primers amplifying both recombinant arms. PCR products were sequenced to further validate correct insertion. Correctly targeted colonies were injected into blastocysts to obtain the chimeric mice. The resulting high-percentage chimeric male was crossed to female C57BL/6J mice to obtain the heterozygous of Cre-dependent Cas9 mice (Rosa26-LSL-Cas9-tdTomato/+). Rosa26-LSL-Cas9-tdTomato/+ mice intercross to obtain the homozygous of Rosa26- LSL-Cas9-tdTomato mice. The homozygous Rosa26-LSL-Cas9-

tdTomato mice were crossed with α-MHC-Cre transgenic mice, generating myocardial Rosa26-Cas9-tdTomato mice and used in later experiments.

EdU administration *in vitro* and *in vivo*

In the *in vitro* experiment, for the 1-day-old and 7-day-old CMs, after transfection for 24 h, the culture medium was replaced by fresh medium for 24 h. That is, 48 h after the cultured cells were seeded, 10 μmol/L EdU was added for 24 h. Cells were fixed at 72 h after seeding and processed for immunofluorescence. Adult CMs were isolated from adult mice that were transduced AAV9-mediated Snhg1 or NC. EdU was administered intraperitoneally (50 mg/kg) at 12, 13 days after transduction. CMs were isolated and processed for immunofluorescence 14 days after transduction.

For the *in vivo* experiment, the P1 mice received EdU (50 mg/kg) by intraperitoneal daily injections at P5 and P6. The hearts of the injected mice were collected 7 days after transduction. In P7 and adult mice, EdU was administered intraperitoneally (50 mg/kg) at 12, 13 days after transduction, and the hearts were collected 14 days after transduction. In adult mice, EdU was administered intraperitoneally (50 mg/kg) at 12, 13 days after transduction, and the hearts were collected 14 days after transduction.

Immunofluorescence analysis

For *in vitro* cultured CMs, the culture medium was washed with PBS. The cells were fixed with 4% paraformaldehyde (Leagene), permeabilized with 0.2% Triton X-100 PBS, and blocked with PBS containing 1% BSA. The cells or slides were incubated with primary

antibodies, including cardiac troponin T (Abcam, ab33589), PCM1 (Abcam, ab72443), vimentin (Abcam, ab8978), Ki67(Abcam, ab15580), pH3 (Abcam, ab170904), Aurora B (Abcam, ab2254), N-cadherin (Abcam, ab18203) and Anillin (Santa Cruz Biotechnology, sc-271814) for 2 h at room temperature, followed by incubation with goat anti-mouse IgG/Alexa Fluor 488 or goat anti-rabbit IgG/Alexa Fluor 555 secondary antibodies (Biosynthesis, bs-0296GA488, bs-0295G-AF555) for 1 h at room temperature. The cells or slides were washed and incubated with DAPI (BioWorld, St. Louis Park, MN, USA). CM borders were defined by staining of tissue with WGA conjugated to Alexa Fluor 555 (Invitrogen) in PBS. Image acquisition was performed with an LSM 880 confocal microscope (Zeiss, Oberkochen, Germany). The Click-iT® EdU Imaging Kits (Life Technologies, USA) to detect EdU incorporation were used according to the manufacturer's instructions. Finally, cells were stained with DAPI.[3-5]

For the *in vivo* experiments, formalin-fixed tissue slides were deparaffinized, and antigen retrieval was performed by microwaving the slides in citrate buffer (0.1 mol/L, pH 6.0) for 14 min. When indicated, the cells or slides immunostained with EdU were further processed using a Click-iT EdU Alexa Fluor 555 Imaging Kit (Invitrogen) to reveal EdU incorporation according to the manufacturer's instructions. Slides were processed for immunofluorescence as described above for the cultured cells.

Cardiomyocytes binucleation assay

Insolated P7 CMs transduced Adv-Snhg1 were cultured with the medium containing 20 μmol/L EdU for 24 h. CMs were washed twice to remove the EdU and cultured for another 24 h in neonatal CM medium with 10% FBS. CMs were harvested at 24 h and 48 h

respectively. the harvested CMs were marked with N-cadherin (Abcam, ab18203) ， cardiac troponin T (Abcam, ab33589) and The Click-iT® EdU Imaging Kits (Life Technologies, USA), and then detected the number of EdU-positive binuclear CMs. Binucleation index were calculated from EdU-positive binuclear CMs in 24 h and 48 h.

Cardiomyocytes, fibroblasts and endothelial cells co-culture

Cell co-culture was performed using a double-chamber co-culture system culture in which adenovirus infected fibroblasts or endothelial cells were cultured in the upper chamber, and CMs were cultured in the lower chamber. To assaying proliferation of the lower chamber CMs, the same amount of CMs were plated in the lower chamber with a 0.4 µm pore size membrane (12-well insert, BD Biosciences) separating the upper and lower chamber cells. After 24 h co-culture, CMs were taken out to assay by Immunofluorescence.

In situ hybridization (ISH)

ISH was performed utilizing the Panomics QuantiGene ViewRNA ISH tissue assay (Affymetrix, Santa Clara, CA, USA) as previously described.[1] Mouse hearts were fixed in 10% formaldehyde and embedded in paraffin. Five-micron sections were cut, deparaffinized, boiled in pretreatment solution, and digested with proteinase K. Heart sections were hybridized for 3 h at 37 ℃ with a custom designed probe against Snhg1 (Affymetrix). Bound probes were amplified according to the protocol from Panomics using PreAmp and Amp molecules. Working label probe oligonucleotides conjugated to 6-AP were added. AP-Enhancer solution was added to each tissue section after washing.

Working label probe oligonucleotides conjugated to 1-AP were added. Slides were counterstained with hematoxylin. Images were acquired with a Nikon Eclipse TE2000-S microscope (Nikon, Tokyo, Japan).

RNA Fluorescent in Situ Hybridization (RNA-FISH)

Isolated CMs grown on coverslips were fixed in 4% paraformaldehyde, and frozen sections (5 μm) of hearts were fixed with 95% ethanol. Then, the cells or the tissue slides were washed 3 times with PBS. The samples were permeabilized in 0.2% Triton X-100 PBS and washed with PBS for 3 times. Then the samples were refixed with 4% paraformaldehyde for 10 min and dehydrated through sequential 5-minute incubations in ethanol (70%, 80%, 95%, 100%). The samples were incubated with prehybridization solution for 30 min and hybridized with hybridization solution and a labeled Snhg1 probe overnight at 42 ℃. The sequences of Snhg1 probe were: 5'-AAAACGTGTTATTTGTAAAATTGAACAGGCCTGGCTCCAAAGTGTAAA-3'. Next, the samples were washed with 50% formamide/2×SSC, 0.1% NP40/1×SSC, 0.5×SSC and 0.2×SSC and blocked with PBS containing 1% BSA. The samples were incubated with primary antibodies, including cardiac troponin T (Abcam), α-SMA (Abcam), CD31 (Abcam), and vimentin (Abcam) for 2 h at room temperature, followed by incubation with goat anti-rabbit IgG/Alexa Fluor 594 secondary antibodies (Biosynthesis) for 1 h at room temperature. The cells or slides were washed and incubated with DAPI. Image acquisition was performed with an LSM 880 confocal microscope.

Stereological analysis

Left ventricles (including the septum) were sampled as previously described.[6, 7] Briefly, heart tissues were embedded in 8% gelatin, and isectors were used to obtain an isotropic, uniform, random alignment of the samples with a maximum diameter of 4 mm. These isectors were used for stereological analysis. An anti-PCM1 antibody was applied to label CM nuclei. WGA was added to identify the cell borders. A minimum of 3-4 isectors were stained, and a minimum of 200 nuclei per animal were counted (nearly 2% of the area of the region of interest). CMs were cut along their longitudinal axis to determine the number of nuclei per cell. The two-step NVX vancomycin-resistant Enterococcus faecium (VREF) method was utilized to estimate the total numbers of nuclei in the heart, as previously described. NV was an estimate of the numerical CM density, and VREF is the reference left ventricle volume. The total number of CMs was calculated based on the number of CM nuclei and the multinucleation level. The analysis was performed by confocal laser scanning microscopy (Carl Zeiss).

Estimation of total number of CMs *in vivo*

As previously described, 8 tissue pieces (1-2 mm diameter) from the left ventricle were sampled. CM nuclei were stained with antibodies against PCM-1, and nuclei were stained with DAPI. The cytoplasm of CMs was stained with cTnT. To facilitate the identification of the cell borders, WGA was added. Using the CAST software, the serial sections could be analyzed all together. In each block, the number of CMs was calculated, and serial

sections were counted to estimate the total number of CMs. For each animal, 4 different tissue blocks were analyzed.

Flow cytometry

After transfected with siRNAs or adenovirus, isolated CMs were cultured for 48 h and then collected and fixed with cold 70% ethanol. Samples were centrifuged for 15 min at ×1200 g. Cell pellets were re-suspended in FxCycle PI/RNase Staining Solution (Thermo Fisher Scientific) and analyzed on MoFlo XDP (Cell Sorter).

Time-lapse videos

After the P7 CMs were transduced with Adv-Snhg1 or Adv-NC for 48 h, the CMs were labeled with tetramethylrhodamine ethyl ester (TMRE), a fluorescent dye that labels mitochondria. Then, the images were imaged for 48 h at 10 min intervals. Live-cell imaging was performed using a Delta Vision Elite system (Applied Precision) on an Olympus IX71 in verted microscope, running Soft WorX6.0. Time-lapse imaging was carried out for 48 h at 10 min intervals, and acquired at a 10x magnification (10/0.3 NA objective) with a Cool Snap HQ2CCD (charge-coupled device) camera (Roper Scientific).

For the P7 CMs isolated from the MYH6-mCherry transgenic mice, after transduction with Adv-Snhg1 for 48 h cells were imaged for 48 h at 10 min intervals. Live-cell imaging was performed using a Delta Vision Elite System (Applied Precision), on an Olympus IX71 in verted microscope, running Soft WorX6.0. Time-lapse imaging was carried out for 48 h at 10 min intervals, and acquired at 10 magnifications (10/0.3 NA objective) with a Cool Snap

HQ2CCD (charge-coupled device) camera (Roper Scientific).

RNA-seq analyses

RNA-seq and genome-wide transcriptome analyses. RNA was collected from the hearts of mice injected with AAV9-Snhg1 or AAV9-NC. RNA-seq experiments were performed by GENE DENOVE (Beijing, China). Briefly, the total RNA was isolated from fresh ventricular tissue using TRIzol (Invitrogen). The RefSeq and Ensembl transcript databases were used as the annotation references for mRNA analyses. The clustering of the index-coded samples was performed on a cBot Cluster Generation System using the TruSeq PE Cluster Kit v3-cBot- HS (Illumina) according to the manufacturer's instructions. Analysis of differential expression was performed using the edgeR R package (3.12.1). The P values were adjusted using the Benjamini and Hochberg method. GO and KEGG pathway analyses were implemented using the cluster Profiler R package. The hierarchical clustering heat map was generated with the ggplot library.

Pull-down assay

The probes of Snhg1 and its antisense RNA for the RNA pull-down assay were designed by Gzscbio Co., Ltd. (Guangzhou, China). The probes for the DNA pull-down assay were synthesized by Gzscbio Co., Ltd. Isolated CMs were washed in ice-cold PBS, lysed in 0.5 mL co-IP buffer, and incubated with 3 μg biotinylated DNA oligo probes against the Snhg1 back-splice sequence at room temperature for 4 h. Next, the CMs were incubated in 50 μL washed streptavidin-coated magnetic beads (Invitrogen) at room temperature for another

hour. RNase-free BSA and yeast tRNA (Sigma) were used to prevent the nonspecific binding of RNA and protein complexes. RNA bound to beads was extracted with TRIzol, while the bound protein was analyzed by Western blotting. The specific bands were extracted and then analyzed by mass spectrometry or Western blotting.

RIP

A biotin-labeled anti-PTEN antibody (1:1000, #4370, cell signaling technology,) and IgG antibodies were added to cell extracts and incubated overnight at 4 °C. Streptavidin-coated magnetic beads were then added and incubated for 4 h at 4 °C. Magnetic beads were pelleted, washed, and resuspended in 1 mL TRIzol. Isolated RNA was reverse transcribed to cDNA and then analyzed by RT-qPCR. The PCR cycling parameters were as follows: an initial denaturation of 95°C for 5 min followed by 40 cycles of 94 °C for 10 s and 65 °C for 40 s, with a final extension at 72 °C for 10 min.

Whole Genome Resequencing

Genome DNA was collected from the hearts of cas9 mice injected with Adv-sgRNA. 30×DNA-seq experiments were performed by BGI Genomics (Shenzhen, China). Briefly, 200 mg fresh ventricular tissue was incubated with lysis buffer containing proteinase K, hen was centrifuged to obtain supernatant. The supernatant was extracted vigorously with phenol/chloroform/isopentanol followed by centrifuging for 10 min. The supernatant was gently mixed with isopropanol and sodium acetate trihydrate. The mix was put at -20 ℃ for two hours and then was centrifuged for 20 min and the pellet was washed by 75%

ethanol. Remove the ethanol by centrifuging and air-dry the pellet for several minutes. The pellet was dissolved by 30-200uL TE for further study.

After DNA extraction, genomic DNA was randomly fragmented to an average size of 200-400 bp. Selected fragments were end repaired and 3' adenylated, then the adaptors were ligated to the ends of these 3' adenylated fragments. The products were amplified by PCR and purified by the Agencourt AMPure XP-Medium kit. The purified double stranded PCR products were heat denatured to single strand, and then circularized by the splint oligo sequence. The single strand circle DNA (ssCirDNA) were formatted as the final library and qualified by QC. The final qualified libraries were sequenced by BGISEQ-500. ssCirDNA molecule formed a DNA nanoball (DNB) containing more than 300 copies through rolling-cycle replication. The DNBs were loaded into the patterned nanoarray by using high density DNA nanochip technology. Finally, pair-end 100 bp reads were obtained by combinatorial Probe-Anchor Syntpesis (cPAS).

The sequencing data was aligned to mouse reference sequences using Burrows-Wheeler Aligner (BWA) tool.[8] The region included 100 nucleotides upstream and downstream of the sgRNA binding site was analyzed to detected nucleotides deleption. Accordingly, the frequencies of nucleotides deleption is calculated to obtain the knockout efficiency of the sgRNA.

Echocardiography

To evaluate cardiac function and dimensions, transthoracic two-dimensional echocardiography was performed on mice anesthetized with 2% isoflurane using a Vevo 2100 Imaging System (Visual Sonics, Ontario, Canada) equipped with a 40-MHz probe.

M-mode tracings in the parasternal short axis view were used to measure the left ventricular internal diameter at end-diastole (LVEDd) and end-systole (LVESd), which were used to calculate left ventricular fractional shortening (LVFS) and the left ventricular ejection fraction (LVEF).

Tissue collection

Mice were anesthetized with 2% isoflurane and then sacrificed by injection of 10% KCl. The hearts and lungs were excised, briefly washed with 0.9% NaCl, weighed, and fixed in 10% formalin at room temperature. The hearts were embedded in paraffin and further processed for histology or immunofluorescence analysis.

Masson's trichrome staining

Formalin-fixed, paraffin-embedded heart tissue slides were deparaffinized via xylene and rehydrated through sequential incubations in ethanol (100%, 100%, 90%, 80%, and 70%) and water. The slices were incubated in Weigert hematoxylin iron for 5 min, differentiated in hydrochloric acid (HCl)-ethanol, incubated in ponceau acid fuchs for 5 min, phosphomolybdic acid for 5 min, and aniline blue or light green (Leagene, Beijing, China) for 5 min. The fibrotic area was measured as the percentage of the total left ventricular area showing fibrosis and quantified with ImageJ software (NIH, Bethesda, MD, USA).

Immunohistochemistry

The MI heart sections were incubated with antibodies against CD31 (Abcam, Cambridge,

UK) at 4 °C overnight, and then with secondary antibodies at 4 °C for 1 h and detected with 3, 3′-diaminobenzidine. The sections were counterstained with hematoxylin. Immunohistochemistry images were captured with an Olympus BX51 microscope (Tokyo, Japan).

RNA isolation and RT-qPCR

Total RNA from isolated CMs or dissected ventricular heart tissue samples was extracted using the E.Z.N.A. ® Total RNA Kit II (Omega Biotek, Norcross, GA, USA) according to the manufacturer's instructions. Cytoplasmic and nuclear RNAs were separated with an RNeasy Midi Kit (Qiagen, Hilden, Germany). The RNAs were treated with DNase I (Invitrogen) to prevent DNA contamination. cDNA was reverse-transcribed from 1 μg of total RNA using the PrimeScript™ RT Master Mix (TaKaRa, Shiga, Japan). For reverse transcription of Snhg1, strand-specific primers and a reverse primer of Snhg1 were used as reverse transcription primers. RT-qPCR was performed with SYBRs Premix Ex Taq™ Kit (TaKaRa) on a Lightcycler 480 (Roche, Basel, Switzerland). Briefly, the 20μl reaction mixtures were incubated at 95 °C for 30 s for the initial 3 denaturation, followed by 40 cycles at 95 °C for 5 s and 60 °C for 34 s. β-actin was used as a housekeeping control gene to normalize gene expression using the $^{\Delta\Delta}$Ct method. All primers were designed by Sangon Biotech (Shanghai, China). Primer sequences are shown in Table S1.

Western blotting

Isolated mouse CMs or dissected mouse ventricular heart tissue samples were lysed in ice-

cold radio immunoprecipitation assay buffer (Ding guo Chang sheng, Beijing, China) with protease inhibitors and phosphatase inhibitors. Protein concentrations were determined with the BCA Protein Quantitative Analysis Kit (Fudebio-tech, Hangzhou, China). Standard Western blotting were performed.[9] Briefly, protein samples were separated by 8-12% sodium dodecyl sulfate-polyacrylamide gel electrophoresis (SDS-PAGE) and transferred onto polyvinylidenedifluoride membranes (Millipore, Billerica, MA, USA). The membranes were incubated at room temperature for 2 h in blocking buffer (5% BSA in Tris-buffered saline and Tween 20 [TBST] buffer). After blocking, the membranes were incubated with primary antibodies overnight at 4 °C. Primary antibodies against the following proteins were used: PTEN (Cell Signaling Technology, #9188), p-Akt (Cell Signaling Technology, #4060), Akt (Cell Signaling Technology, #9272), pGsk3β (Abcam, ab131097), c-Myc (Cell Signaling Technology, #18583,Proteintech, 10828-1-AP),Vegfa (Proteintech, 66828-1-Ig), Gapdh (Biosynthesis, bs-2188R) and β-actin (Biosynthesis, bs-0061R). After the membranes were washed three times with TBST, they were incubated with donkey anti-rabbit IgG (Abcam, ab16284) for 1 h at room temperature. The membranes were developed with the electrochemiluminescence method according to the manufacturer's instructions (Millipore) and detected on a chemiluminescence imaging GeneGnome XRQ System (Syngene, Bangalore, India). To calculate the relative density, ImageJ software was used, and the intensity of each band was normalized to that of β-actin.

Luciferase assay

Luciferase assays were conducted with the Dual-luciferase Reporter Kit (Promega). The

pRL vector constitutively expressing Renilla luciferase was used to normalize for transfection efficiency. A total of 2 × 10^5 CMs were plated in 12-well dishes 24 h before transfections. On the day of transfection, each well was transfected with pRL, pGL3 Basic (to assess basal reporter activity) or Myc promoter-pGL3 and the indicated plasmids. Twenty-four hours later, luciferase activity was measured using the Wallac 1450 MicroBeta TriLux System (Perkin Elmer). Experiments were carried out 3 times in triplicates, and error bars represent SD.

References

1. Huang S, Li X, Zheng H, Si X, Li B, Wei G, Li C, Chen Y, Chen Y, Liao W, Liao Y, Bin J. Loss of Super-Enhancer-Regulated circRNA Nfix Induces Cardiac Regeneration After Myocardial Infarction in Adult Mice. Circulation 2019;**139**(25):2857-2876.

2. Eulalio A, Mano M, Dal Ferro M, Zentilin L, Sinagra G, Zacchigna S, Giacca M. Functional screening identifies miRNAs inducing cardiac regeneration. Nature 2012;**492**(7429):376-81.

3. Li B, Hu Y, Li X, Jin G, Chen X, Chen G, Chen Y, Huang S, Liao W, Liao Y, Teng Z, Bin J. Sirt1 Antisense Long Noncoding RNA Promotes Cardiomyocyte Proliferation by Enhancing the Stability of Sirt1. J Am Heart Assoc 2018;**7**(21):e009700.

4. Chen Y, Li X, Li B, Wang H, Li M, Huang S, Sun Y, Chen G, Si X, Huang C, Liao W, Liao Y, Bin J. Long Non-coding RNA ECRAR Triggers Post-natal Myocardial Regeneration by Activating ERK1/2 Signaling. Mol Ther 2019;**27**(1):29-45.

5. Li X, He X, Wang H, Li M, Huang S, Chen G, Jing Y, Wang S, Chen Y, Liao W, Liao Y, Bin J. Loss of AZIN2 splice variant facilitates endogenous cardiac regeneration.

Cardiovascular research 2018;**114**(12):1642-1655.

6. Alkass K, Panula J, Westman M, Wu TD, Guerquin-Kern JL, Bergmann O. No Evidence for Cardiomyocyte Number Expansion in Preadolescent Mice. Cell 2015;**163**(4):1026-36.

7. Bruel A, Nyengaard JR. Design-based stereological estimation of the total number of cardiac myocytes in histological sections. Basic Res Cardiol 2005;**100**(4):311-9.

8. Li H, Durbin, R. Fastand accurate long-read alignment with burrows-wheeler transform. Bioinformatics, 2010, **26**:589-595.

9. Chakraborty S, Sengupta A, Yutzey KE. Tbx20 promotes cardiomyocyte proliferation and persistence of fetal characteristics in adult mouse hearts. J Mol Cell Cardiol 2013;**62**:203-13.

Supplemental figure legends

Figure S1. Snhg1 is highly expressed in neonatal hearts and conserved across mammalians.

(A) RT-qPCR analysis of Snhg1, 3, 4, 5, 7, 8, 9, 12, 15, 17, 18, and 20 in P1 and adult mouse hearts as detected by RT-qPCR, n=5. (B) Conservation analysis of Snhg1. (C) RT-qPCR analysis of Snhg1 levels in P1 mouse heart, liver, muscle, skin, kidney, and brain tissues, n=5. (D) Detection of Snhg1 expression in adult heart tissue after MI by *ISH*, 1-3 respectively indicated remote zone, border zone and infarcted zone, n=5. All data are expressed as the mean ± SD, $^{*}P<0.05$ using t-tests in A, one-way ANOVA test followed by LSD post hoc test in C and D.

Figure S2. Adenovirus-mediated Snhg1 overexpression promotes P7 CM proliferation *in vitro*.

(A) The purity of isolated P7 CMs is approximately 80%, and isolated CMs were stained with cTnT, PCM-1, and DAPI, 751 cells from 5 mice. (B) GFP/cTnT double-staining in P7 CMs after transduction with PBS or Adv-Snhg1-GFP, 638 CMs from 5 mice in Adv-Snhg1-GFP group. (C) Quantification of Snhg1 expression by RT-qPCR in isolated P7 CMs transduced with Adv-NC or Adv-Snhg1, n=5. (D) Immunostaining of Anilin for P7 CMs transduced with Adv-Snhg1, PCM1 represent cardiomyocyte nuclei, 433 CMs from 5 mice in Ad-NC group, 503 CMs from 5 mice in Ad-Snhg1 group. (E) Western blotting analysis and quantification of pH3 and Aurora B protein levels in isolated P7 CMs transduced with Adv-NC or Adv-Snhg1. β-actin was used as a loading control, n=5. (F) the proportion of GFP+ cell in the EdU+ cardiomyocytes. 657 CMs from 5 mice, Arrow, GFP+ CMs, Triangle, GPP- CMs. (G) the proportion of GFP+ cell in the pH3+ cardiomyocytes. 574 CMs from 5 mice, Arrow, GFP+ CMs. (H) Quantification of the CM number after injected with Adv-NC or Adv-Snhg1, 545 CMs from 5 mice in Ad-NC group, 623 CMs from 5 mice in Ad-Snhg1 group. (I) Schematic of the CM binucleation assay after Adv-Snhg1 infection. Isolated CMs are treated with EdU for 24 h. EdU is then washed

out and CMs are cultured for another 24 h to complete one cell cycle. All data are expressed as the mean ± SD, $^{*}P<0.05$ using t-tests in A-H.

Figure S3. Snhg1 overexpression promotes P7 CM proliferation *in vivo*.

(A) The transduction efficiency of AAV9-Snhg1 was approximately 80%, as revealed by GFP and CM specific marker staining, n=5. (B) Quantification of Snhg1 expression by RT-qPCR in P7 mouse hearts injected with AAV9-NC or AAV9-Snhg1, n=5. (C) ISH assays confirmed that Snhg1 was significantly increased in P7 mouse hearts injected with AAV9-Snhg1 or AAV9-NC. the brown dot cluster indicates Snhg1, n=3. (D) Immunostaining and quantification of Aurora B in P7 mouse hearts injected with AAV9-NC or AAV9-Snhg1, n=5. (E) Immunostaining and quantification of pH3-positive CMs in P7 Myh6-mCherry transgenic mice injected with AAV9-NC or AAV9-Snhg1, n=5. All data are expressed as the mean ± SD, $^{*}P<0.05$ using t-tests in A-E.

Figure S4. The effect of Endothelial cells, Fibroblasts and CMs co-culture on CMs proliferation.

(A) Diagrammatic representation of endothelial cells (ECs)/fibroblasts (FBs) and CMs co-culture system. (B) Detection of Ki67+ CMs after co-cultured with FBs infected with Adv-NC or Adv-Snhg1, 473 CMs from 5 mice in CM group, 501 CMs from 5 mice in CM+FB group, 493 CMs from 5 mice in CM+ FB-Adv-NC group, 529 CMs from 5 mice in CM+ FB-Adv-Snhg1 group. (C) Detection of pH3+ CMs after co-cultured with FBs infected with Adv-NC or Adv-Snhg1, 423 CMs from 5 mice in CM group, 478 CMs from 5 mice in CM+FB group, 514 CMs from 5 mice in CM+ FB-Adv-NC group, 539 CMs from 5 mice in CM+ FB-Adv-Snhg1 group. (D) Detection of Ki67+ CMs after co-cultured with ECs infected with Adv-NC or Adv-Snhg1, 457 CMs from 5 mice in CM group, 523 CMs from 5 mice in CM+EC group, 534 CMs from 5 mice in CM+ EC-Adv-NC group, 507 CMs from 5 mice in CM+ EC-Adv-Snhg1 group. (E) Detection of pH3+ CMs after co-cultured with ECs infected with Adv-NC or Adv-Snhg1. All data are expressed as the mean ± SD, $*P<0.05$ using one-way ANOVA test followed by LSD post hoc test in B-E.

Figure S5. Snhg1 regulates human iPS-derived CM and macrophage proliferation, but not fibroblasts proliferation.

(A) EdU immunofluorescence staining in fibroblasts (FBs) transduced with Adv-NC or Adv-Snhg1 and quantification of EdU-positive FBs, n=5. (B) EdU immunofluorescence staining in FBs transfected with si-NC or si-Snhg1 and quantification of EdU-positive FBs, n=5. (C) Representative FACS plots showing infection efficiency of GFP adenovirus in Thy1+ cardiac fibroblasts infected with 10 or 100 MOI, compared to CMs infected with 10 MOI of the adenovirus. (D) Immunostaining of Ki67 for macrophages transduced with Adv-sh-NC or Adv-sh-Snhg1, 785 macrophages from 5 independent experiments in Ad-sh-NC group, 638 macrophages from 5 independent experiments in Ad-sh-Snhg1 group. (E) Immunostaining of pH3 for macrophages transduced with Adv-sh-NC or Adv-sh-Snhg1, 785 macrophages from 5 independent experiments in Ad-sh-NC group, 836 macrophages from 5 independent experiments in Ad-sh-Snhg1 group. (F) Immunocytochemistry of 60-day-old human iPS-derived CMs infected with Adv-NC or Adv-Snhg1 and immunostained 48 h later with antibodies against pH3, cardiac troponin T, and DAPI to mark nuclei, n=5. All data are expressed as the mean ± SD, $^{*}P<0.05$ using t-tests in A, B, D, E and F.

Figure S6. The transduction efficiency of AAV9-Snhg1.

(A) The transduction efficiency of AAV9-Snhg1 in adult CMs was approximately 80% as revealed by GFP and CM specific marker staining, n=5. (B) Representative In vivo bioluminescent images and Bright field captured on day 14 after injection with GFP-labelled AAV9-Snhg1 virus. Square indicates thoracic incision, arrow indicates the heart with GFP fluorescence. (C) GFP/cTnT double-staining in mouse adult hearts injected with PBS or AAV9-Snhg1-GFP, n=5. (D) Quantification of Snhg1 expression by RT-qPCR in adult mouse hearts injected with AAV9-NC or AAV9-Snhg1, n=5. (E) The transduction efficiency of AAV9-Snhg1-GFP in main types of cells in adult heart injected with AAV9-Snhg1-GFP, n=5. All data are expressed as the mean ± SD, $^{*}P<0.05$ using t-tests in A, C and D.

Figure S7. Snhg1 induces adult CM proliferation

(A) Stereological analysis revealed the number of CMs in adult mice injected with AAV9-NC or AAV9-Snhg1. Sections 1-8 out of 8 serial sections are shown, n=5. (B) CM size evaluation by WGA staining in adult mouse hearts after transduction with AAV9-NC or AAV9-Snhg1. (337 CMs from 5 mice in AAV9-NC group, 342 CMs from 5 mice in AAV9-Snhg1). (C) Heart weight (HW) to body weight (BW) ratio of adult hearts injected with AAV9-NC or AAV9-Snhg1, n=5. Morphology (upper panel) and Masson trichrome staining (lower panel) of hearts. All data are expressed as the mean ± SD, $^{*}P<0.05$ using t-tests in B.

Figure S8. Snhg1 improves adult cardiac function post-MI.

(A) The adult mouse MI model was confirmed using an electrocardiogram ST-segment elevation. (B) GFP/cTnT double-staining in remote zone, border zone and infarcted zone of MI mouse adult hearts injected with AAV9-Snhg1-GFP, n=5. (C) Immunostaining and quantification of pH3-positive CMs in P7 Myh6-mCherry transgenic mice injected with AAV9-NC or AAV9-Snhg1, n=5. (D) CM size evaluation by WGA staining in adult infarcted mouse hearts after transduction with AAV9-NC or AAV9-Snhg1. (357 CMs from 5 mice in AAV9-NC group, 362 CMs from 5 mice in AAV9-Snhg1 group). (E) Heart weight (HW) to body weight (BW) ratio of adult infarcted hearts injected with AAV9-NC or AAV9-Snhg1. Morphology (upper panel) and Masson trichrome staining (lower panel) of hearts. All data are expressed as the mean ± SD, $^{*}P<0.05$ using t-tests in C-D.

Figure S9. Snhg1 improves adult cardiac function in the I/R mouse model.

(A-B) Immunofluorescence and quantification of EdU and pH3 for AAV9-NC and AAV9-Snhg1-injected adult mouse hearts 14 days after I/R, positive CMs are indicated by arrows, n=6. (C) Immunofluorescence staining and quantification of TUNEL-positive CMs in the AAV9-NC and AAV9-Snhg1-injected adult mouse hearts 14 days after I/R. TUNEL-positive CMs are indicated by arrows, n=6. (D) Immunofluorescence and quantification of IB4-positive cells for AAV9-NC and AAV9-Snhg1-injected adult mouse hearts 14 days after I/R. IB4-positive cells are indicated by arrows, n=5. (E) Representative images of

Masson's trichrome staining and immunohistochemistry for CD31 in AAV9-NC or AAV9-Snhg1-injected adult mice 28 days after I/R and quantification of CD31-positive cells, n=5. (F) Representative images of Masson's trichrome-stained heart sections in Sham or AAV9-NC or AAV9-Snhg1-injected adult mice 28 days after I/R and quantification of infarct size, n=6. (G) Representative images of echocardiography analysis on Sham or AAV9-NC or AAV9-Snhg1-injected adult mouse hearts at 28 days after I/R and quantification of LVESd, LVEDd, LVEF, and LVFS, n=10. All data are expressed as the mean ± SD, $^{*}P<0.05$ using t-tests in A-E, one-way ANOVA test followed by LSD post hoc test in F-G.

Figure S10. Snhg1 depletion decreased neonatal CM proliferation *in vitro*.

(A) RT-qPCR of Snhg1 in isolated P1 CMs transfected with si-NC or si-Snhg1, n=5. (B) Western blotting analysis of pH3 and Aurora B protein levels in isolated P1 CMs transfected with si-NC or si-Snhg1, n=5. All data are expressed as the mean ± SD, $^{*}P<0.05$ using t-tests in A-B.

Figure S11. Snhg1 depletion decreased neonatal CM proliferation *in vivo*.

(A) Schematic of the experiments in 1day mouse hearts injected with adenovirus. IF, immunofluorescence. (B) RT-qPCR of Snhg1 expression in neonatal mouse hearts injected with Adv-sh-NC or Adv-sh-Snhg1, n=5. (C) *ISH* assays of Snhg1 expression in neonatal mouse hearts injected with Adv-sh-NC or Adv-sh-Snhg1, n=5. (D) Immunofluorescence of EdU in neonatal hearts injected with Adv-sh-NC or Adv-sh-Snhg1, n=5. Arrows, positive CMs. (E) Immunofluorescence of Ki67 and pH3 in neonatal hearts injected with Adv-sh-NC or Adv-sh-Snhg1, n=5. Arrows, positive CMs. (F-G) Immunofluorescence of Aurora B-positive CMs in neonatal hearts injected with Adv-sh-NC or Adv-sh-Snhg1, n=5. All data are expressed as the mean ± SD, $^{*}P< 0.05$ using t-tests in B-G.

Figure S12. Generation of myocardial Snhg1-deficient mice using CRISPR-Cas9 technology.

(A) Schematic of sgRNA targeting site at snhg1 locus. The red color represents the sgRNA targeting sequence, and the green color represents the PAM sequence. (B) Immunostaining

of Cas9 in transgenic mouse hearts, α-MHC-Cre- and α-MHC-Cre+ mouse were littermates of generation of α-MHC-Cre transgenic mice and Rosa26-LSL-Cas9-tdTomato. (C) Immunostaining of GFP in cas9-tdTomato mouse hearts at 7 days after injection with Adv -sgRNA (Snhg1)-GFP, n=5. (D) Base percentage composition along reads of the DNA-seq data from the heart of cas9 mouse injected Adv-sgRNA. The A curve overlap with the T curve, and the G curve overlap with the C curve, indicating a balanced base composition of raw reads. (E) Distribution of qualities along reads from the heart of cas9 mouse injected Adv-sgRNA. Each dot in the image represents the quality value of the corresponding position along reads. If the percentage of the bases with low quality (<20) was considered low, then the sequencing quality of this lane was considered good. (F) The knockout efficiency of Snhg1 using sgRNAs in heart of cas9 mouse, knockout efficiency was calculated as the frequencies of nucleotides deleption in sgRNA binding site, n=2. (G) RT-qPCR assays detecting Snhg1 expression in cas9-tdTomato mouse hearts at 7 days after injection with Adv-sgRNA (Snhg1)-GFP or Adv-NC, n=5. (H) Detection of Snhg1 expression in sham operated and infarcted neonatal mouse hearts by using ISH analysis, n=5. All data are expressed as the mean ± SD, $^{*}P< 0.05$ using t-tests in C, G and H.

Figure S13. Next-generation RNA sequencing (RNA-seq) of Snhg1 overexpression and control group from P7 mice

(A) Hierarchical clustering of differentially expressed genes between AAV9-Snhg1 and control mimic-injected P7 hearts. Red and blue colors indicate up-regulated or down-regulated genes. (B) Scatter plot showing top 20 enriched GO terms of biological process for differentially expressed genes between AAV9-Snhg1 and control mimic-injected P7 hearts. (C) RT-qPCR analysis of the expression of differentially expressed genes related to the cell cycle, PI3K-Akt, and Hippo pathways, n=5. All data are expressed as the mean ± SD, $^{*}P< 0.05$ using t-tests in C.

Figure S14. Snhg1 regulates CM proliferation through the PTEN/PI3K-Akt/c-Myc pathway.

(A) NanoLC-MS/MS spectrum of PTEN peptides. (B) Western blotting analysis and quantification of PTEN protein levels in isolated CMs transduced with Adv-NC, Adv-Snhg1, si-NC, or si-Snhg1. β-Actin was used as a loading control, n=5. (C) Western blotting analysis and quantification of PI3K, p-Akt, and Akt protein levels in P7 mice injected with AAV9-NC, AAV9-Snhg1, AAV9-PTEN, and AAV9-Snhg1+AAV9-PTEN. β-Actin was used as a loading control, n=4. (D-E) Immunofluorescence and quantification of Ki67-positive CMs in P7 mice injected with AAV9-NC, AAV9-Snhg1, AAV9-PTEN, or AAV9-Snhg1+AAV9-PTEN, n=5. (F) Western blotting comparing p-AKT, p-GSK3β, and c-Myc levels in P7 CMs treated with si-NC, si-Snhg1, NC or LY294002. β-Actin was used as a loading control, n=4. (G) Western blotting comparing c-Myc levels in P7 CMs treated with NC, si-GSK3β, LY294002, or LY294002+si-GSK3β. β-Actin was used as a loading control, n=4. All data are expressed as the mean ± SD, $^{*}P< 0.05$ using t-tests in B, one-way ANOVA test followed by LSD post hoc test in C and E-G.

Figure S15. Snhg1 promotes angiogenesis by Akt/ GSK3 β /VEGF pathway.

(A) Western blotting analysis and quantification of VEGFA and p-GSK3 β protein levels in endothelial cells treated with Adv-NC, Adv-Snhg1, Adv-NC+LY294002, and Adv-Snhg1+LY294002. n= 5. (B) Western blotting analysis and quantification of VEGFA protein levels in endothelial cells treated with si-NC, si-GSK3β, si-NC+LY294002, and si-GSK3β+LY294002. n= 5. All data are expressed as the mean ± SD, $^{*}P< 0.05$ using one-way ANOVA test followed by LSD post hoc test in A and B.

Figure S16. c-Myc binds to its promoter region of Snhg1.

(A) Construction of Snhg1-WT and Snhg1-MU promoter sequences. (B) Luciferase activity in CMs transfected with luciferase-Snhg1-WT or luciferase-Snhg1-MU after Adv-c-Myc interference. (C) Western blotting analysis and quantification of c-Myc protein levels in cardiomyocyte at different age. n= 5. All data are expressed as the mean ± SD, $^{*}P< 0.05$ using one-way ANOVA test followed by LSD post hoc test in B and C.

Figure S17. Entire unedited gel for all representative cropped gels in the manuscript.

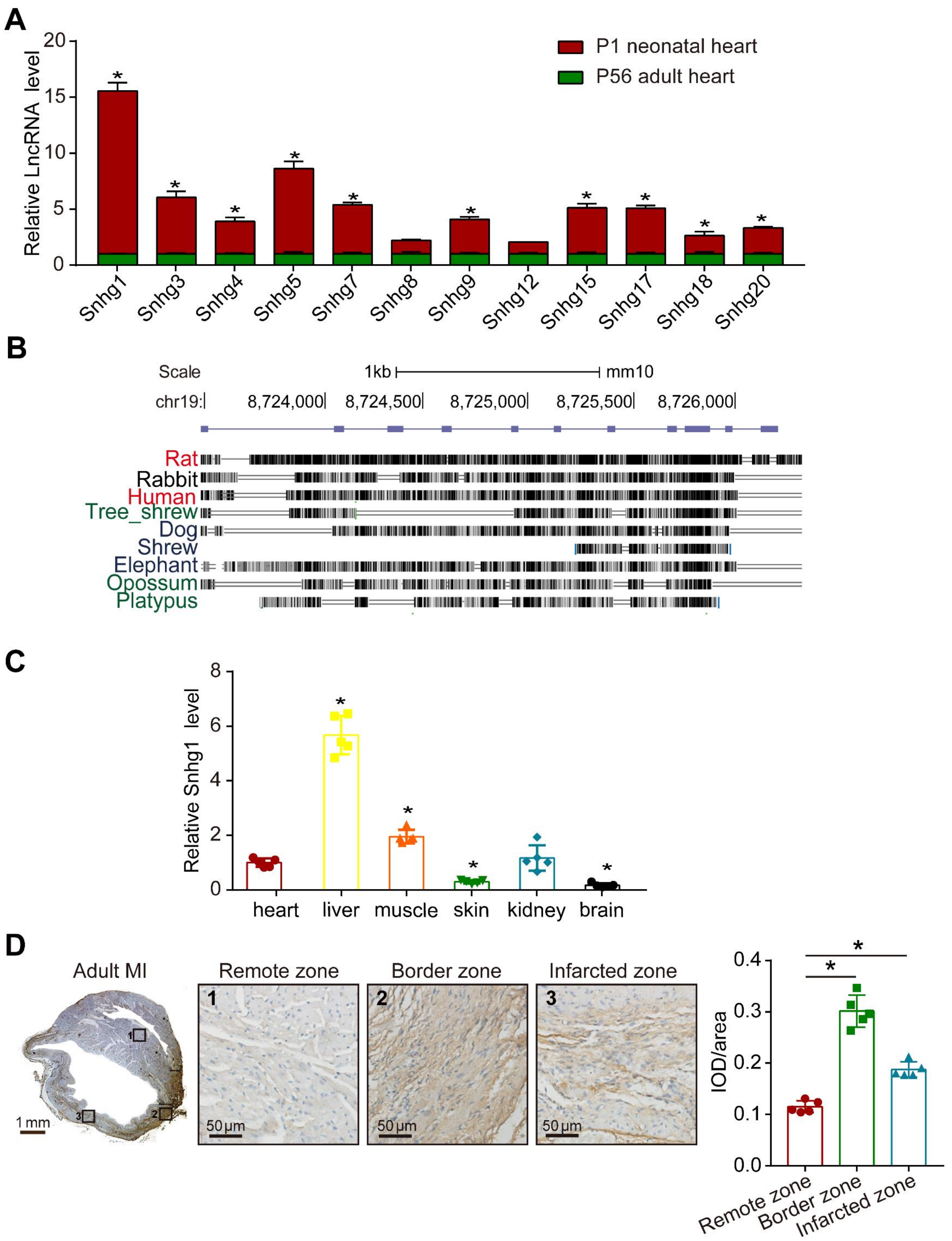
A
Relative LncRNA level
P1 neonatal heart
P56 adult heart
20
15
10
5
0
Snhg1
Snhg3
Snhg4
Snhg5
Snhg7
Snhg8
Snhg9
Snhg12
Snhg15
Snhg17
Snhg18
Snhg20
B
Scale
1kb
mm10
chr19:
8,724,000
8,724,500
8,725,000
8,725,500
8,726,000
Rat
Rabbit
Human
Tree_shrew
Dog
Shrew
Elephant
Opossum
Platypus
C
Relative Snhg1 level
8
6
4
2
0
heart
liver
muscle
skin
kidney
brain
D
Adult MI
Remote zone
Border zone
Infarcted zone
1 mm
50 μm
IOD/area
0.4
0.3
0.2
0.1
0.0
Remote zone
Border zone
Infarcted zone

Figure S1

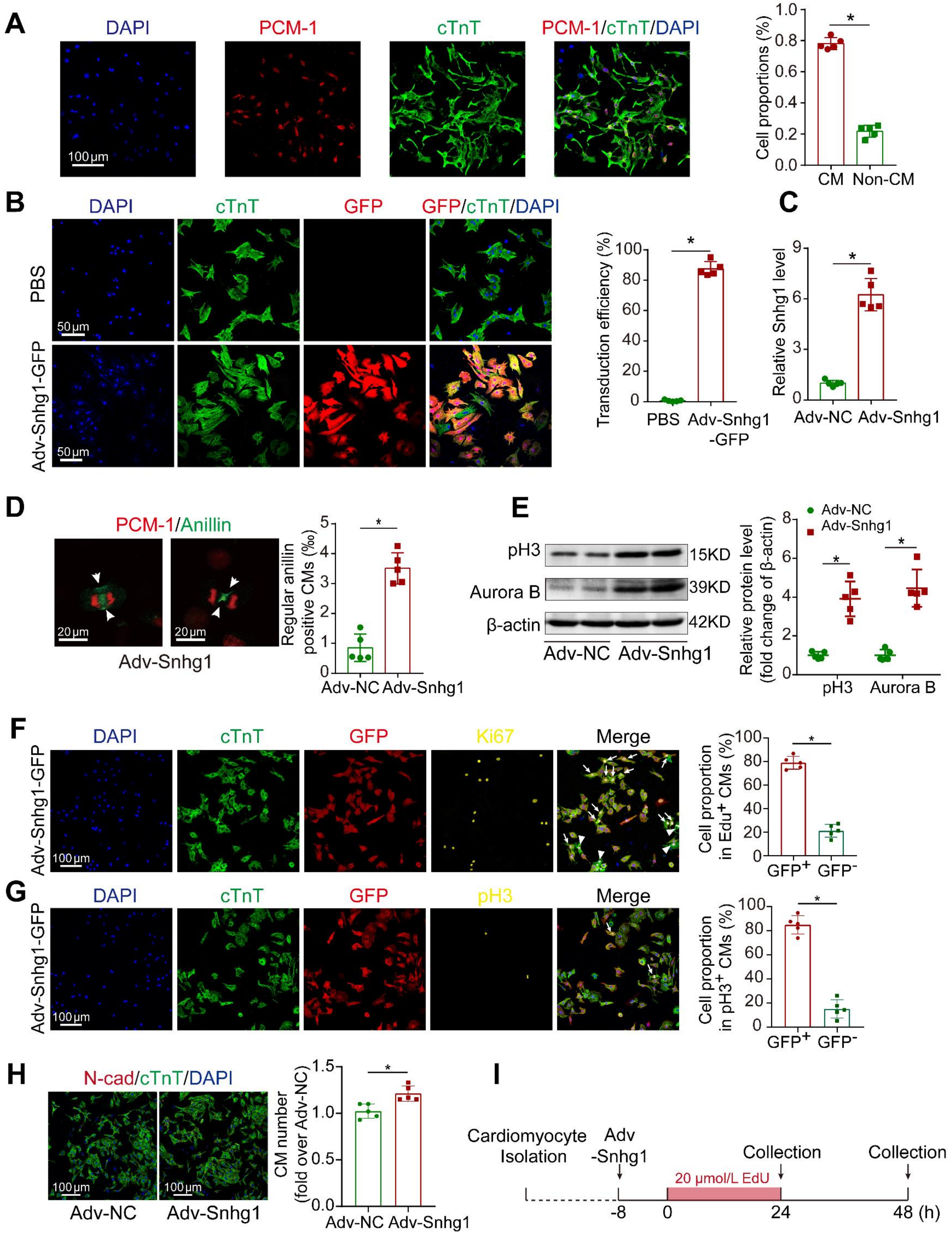
A
DAPI
PCM-1
cTnT
PCM-1/cTnT/DAPI
100 μm
Cell proportions (%)
CM
Non-CM
B
DAPI
cTnT
GFP
GFP/cTnT/DAPI
PBS
Adv-Snhg1-GFP
50 μm
Transduction efficiency (%)
PBS
Adv-Snhg1 -GFP
C
Relative Snhg1 level
Adv-NC
Adv-Snhg1
D
PCM-1/Anillin
20 μm
Adv-Snhg1
Regular anillin positive CMs (‰)
Adv-NC
Adv-Snhg1
E
pH3
Aurora B
β-actin
15KD
39KD
42KD
Adv-NC
Adv-Snhg1
Relative protein level (fold change of β-actin)
F
DAPI
cTnT
GFP
Ki67
Merge
100 μm
Cell proportion in Edu+ CMs (%)
GFP+
GFP-
G
pH3
Cell proportion in pH3+ CMs (%)
H
N-cad/cTnT/DAPI
CM number (fold over Adv-NC)
I
Cardiomyocyte Isolation
Adv -Snhg1
Collection
Collection
20 μmol/L EdU
-8
0
24
48 (h)

Figure S2

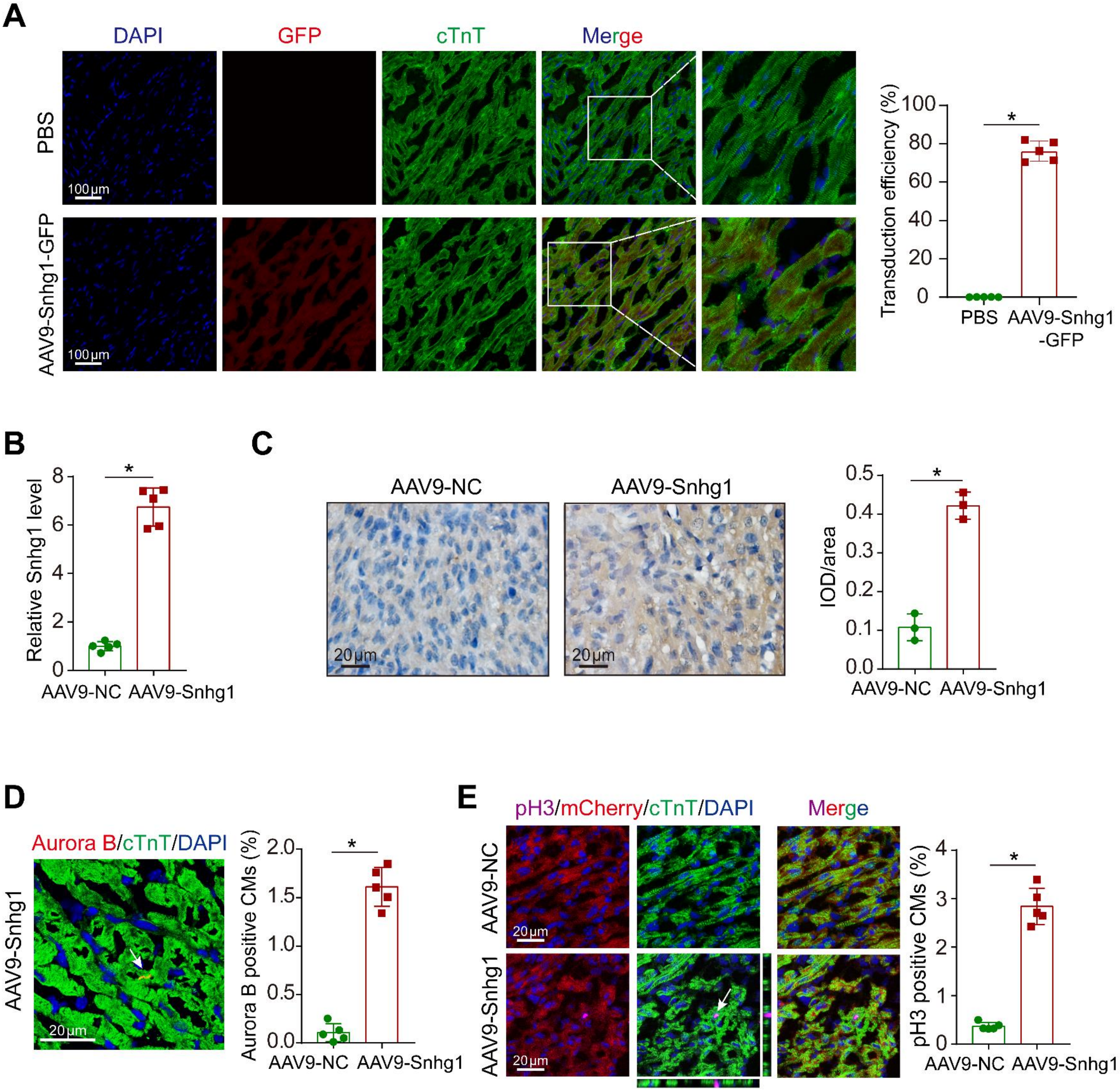
A
DAPI
GFP
cTnT
Merge
PBS
AAV9-Snhg1-GFP
100 μm
Transduction efficiency (%)
100
80
60
40
20
0
PBS
AAV9-Snhg1
-GFP
*
B
Relative Snhg1 level
8
6
4
2
0
AAV9-NC
AAV9-Snhg1
*
C
AAV9-NC
AAV9-Snhg1
20 μm
IOD/area
0.5
0.4
0.3
0.2
0.1
0.0
*
D
Aurora B/cTnT/DAPI
AAV9-Snhg1
20 μm
Aurora B positive CMs (%)
2.0
1.5
1.0
0.5
0.0
*
E
pH3/mCherry/cTnT/DAPI
Merge
AAV9-NC
AAV9-Snhg1
pH3 positive CMs (%)
4
3
2
1
0
*

Figure S3

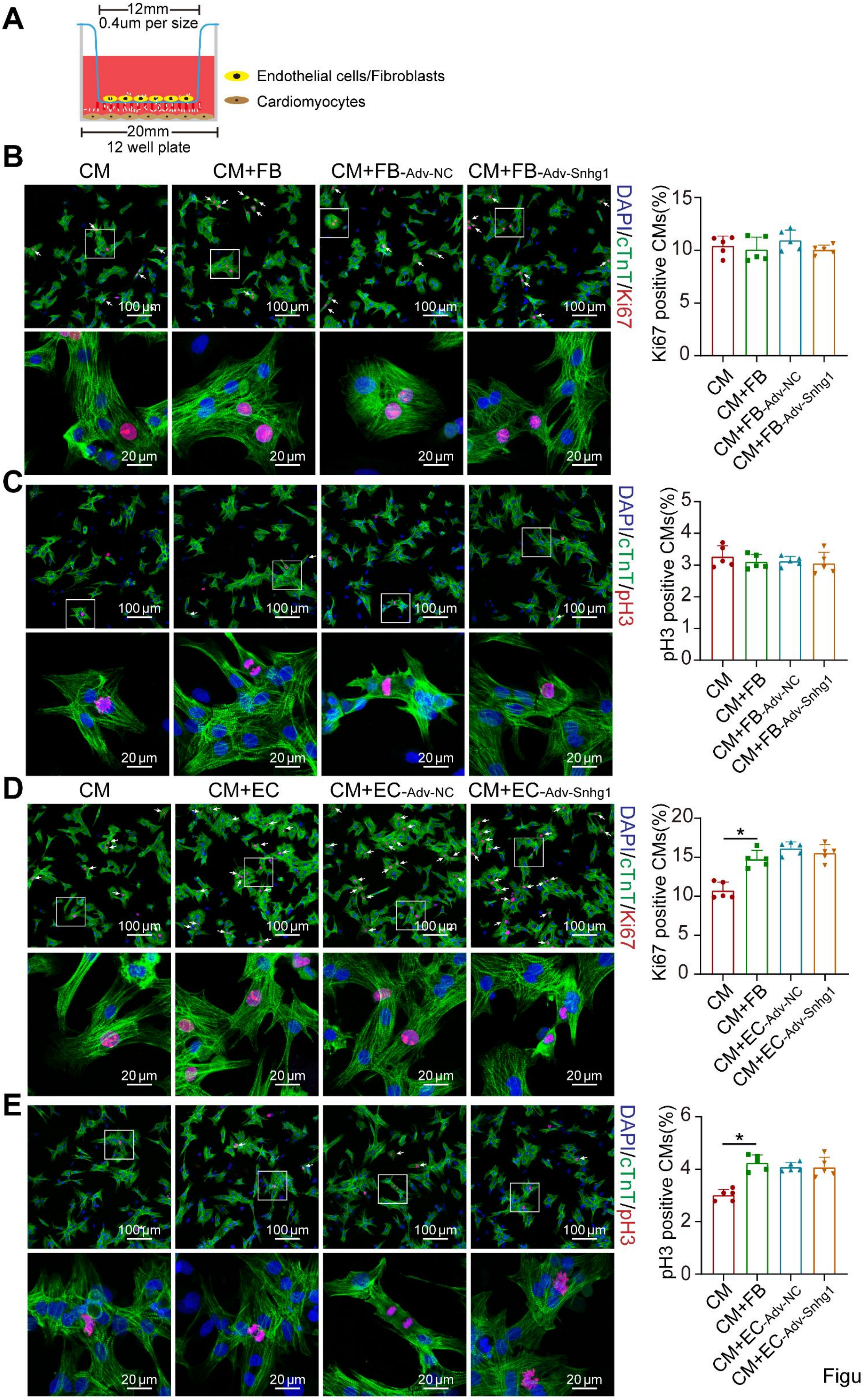
A
12mm
0.4um per size
Endothelial cells/Fibroblasts
Cardiomyocytes
20mm
12 well plate
B
CM
CM+FB
CM+FB-Adv-NC
CM+FB-Adv-Snhg1
DAPI/cTnT/Ki67
Ki67 positive CMs(%)
C
DAPI/cTnT/pH3
pH3 positive CMs(%)
D
CM+EC
CM+EC-Adv-NC
CM+EC-Adv-Snhg1
E
100 µm
20 µm

Figure S4

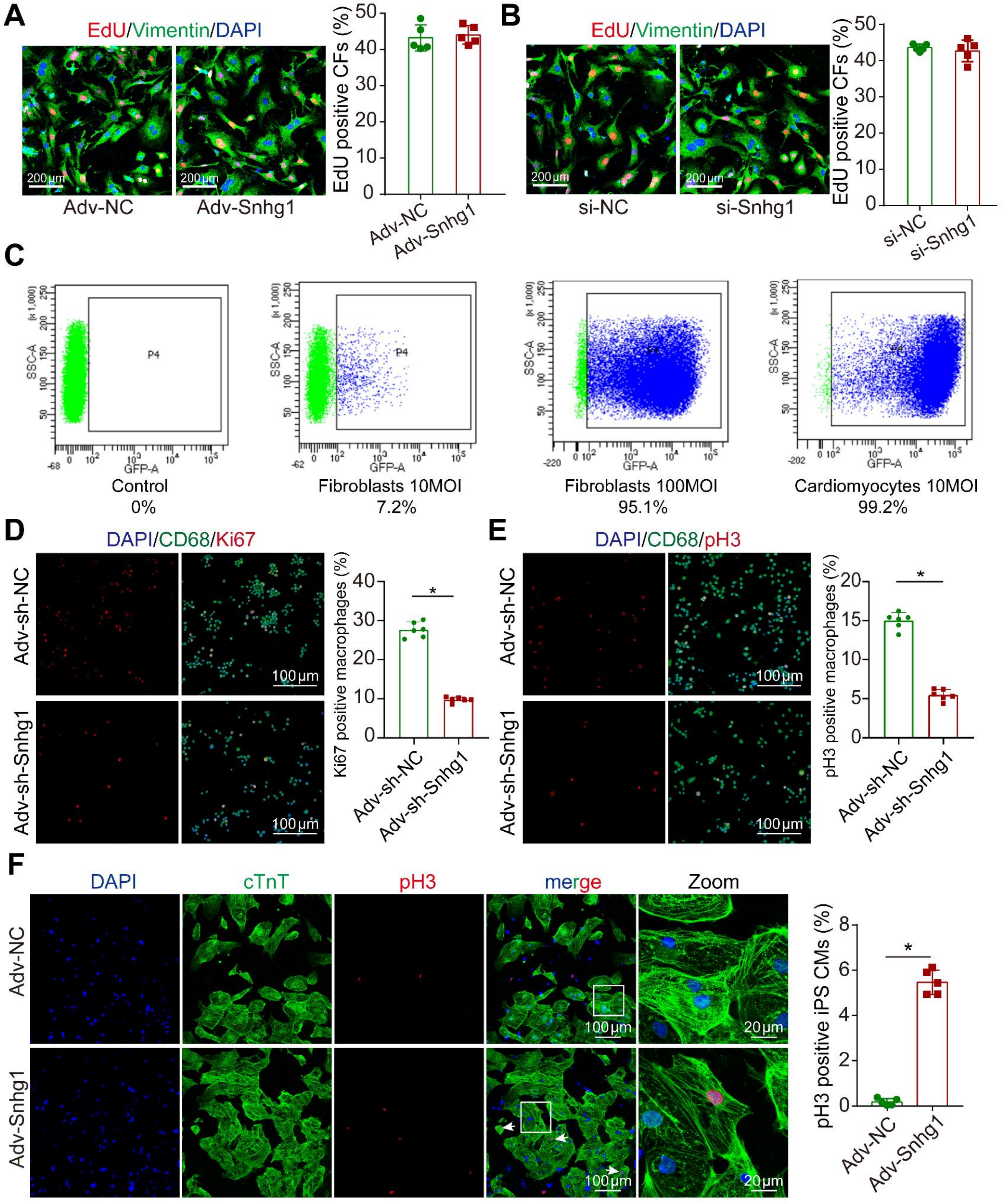
A
EdU/Vimentin/DAPI
200 μm
Adv-NC
Adv-Snhg1
EdU positive CFs (%)
B
si-NC
si-Snhg1
C
SSC-A
GFP-A
P4
Control
0%
Fibroblasts 10MOI
7.2%
Fibroblasts 100MOI
95.1%
Cardiomyocytes 10MOI
99.2%
D
DAPI/CD68/Ki67
Adv-sh-NC
Adv-sh-Snhg1
100 μm
Ki67 positive macrophages (%)
E
DAPI/CD68/pH3
pH3 positive macrophages (%)
F
DAPI
cTnT
pH3
merge
Zoom
20 μm
pH3 positive iPS CMs (%)

Figure S5

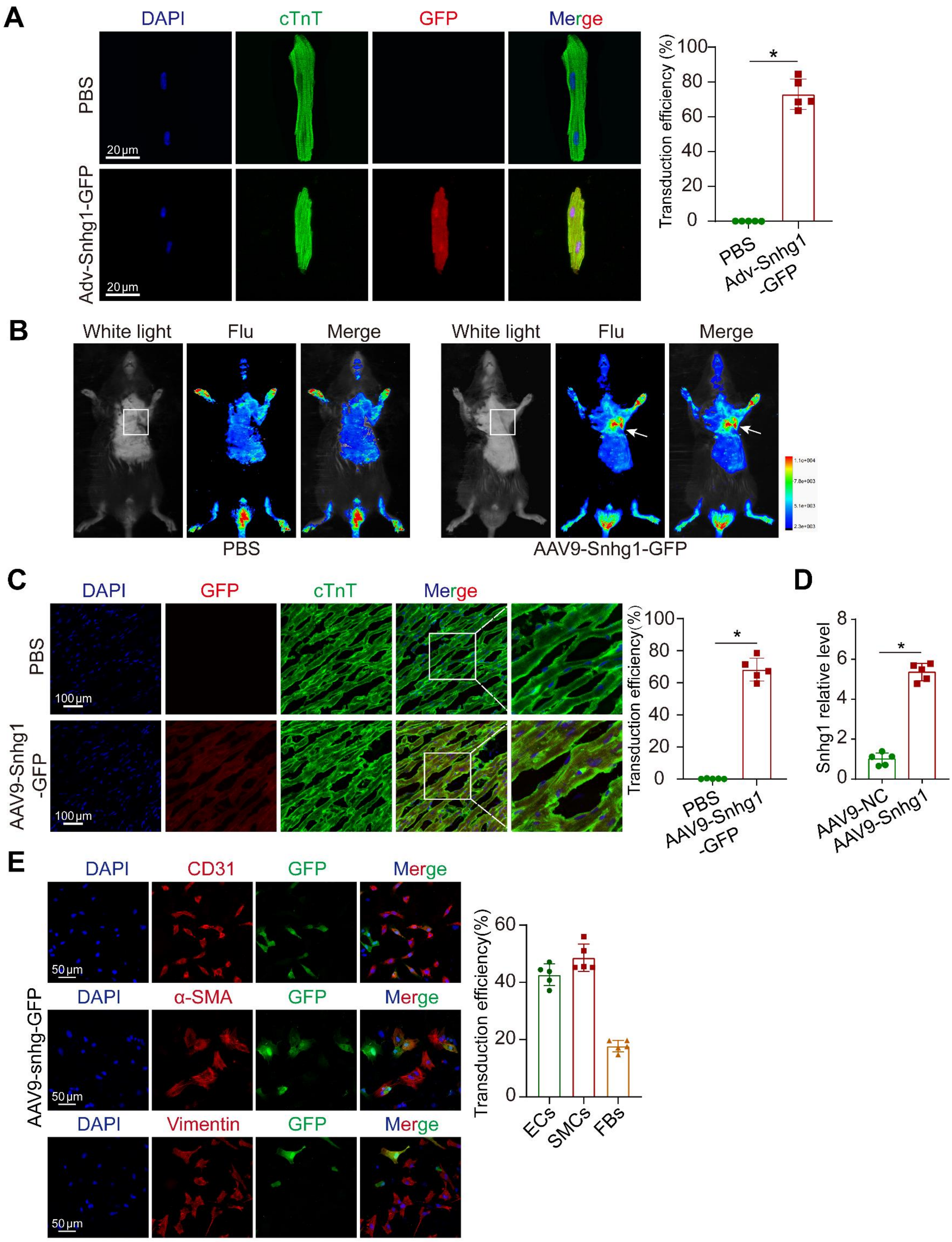

A
DAPI
cTnT
GFP
Merge
PBS
Adv-Snhg1-GFP
20 μm
20 μm
Transduction efficiency (%)
100
80
60
40
20
0
*
PBS
Adv-Snhg1-GFP
B
White light
Flu
Merge
White light
Flu
Merge
PBS
AAV9-Snhg1-GFP
C
DAPI
GFP
cTnT
Merge
PBS
AAV9-Snhg1-GFP
100 μm
100 μm
Transduction efficiency(%)
100
80
60
40
20
0
*
PBS
AAV9-Snhg1-GFP
D
Snhg1 relative level
8
6
4
2
0
*
AAV9-NC
AAV9-Snhg1
E
DAPI
CD31
GFP
Merge
DAPI
α-SMA
GFP
Merge
DAPI
Vimentin
GFP
Merge
AAV9-snhg-GFP
50 μm
50 μm
50 μm
Transduction efficiency(%)
60
40
20
0
ECs
SMCs
FBs

Figure S6

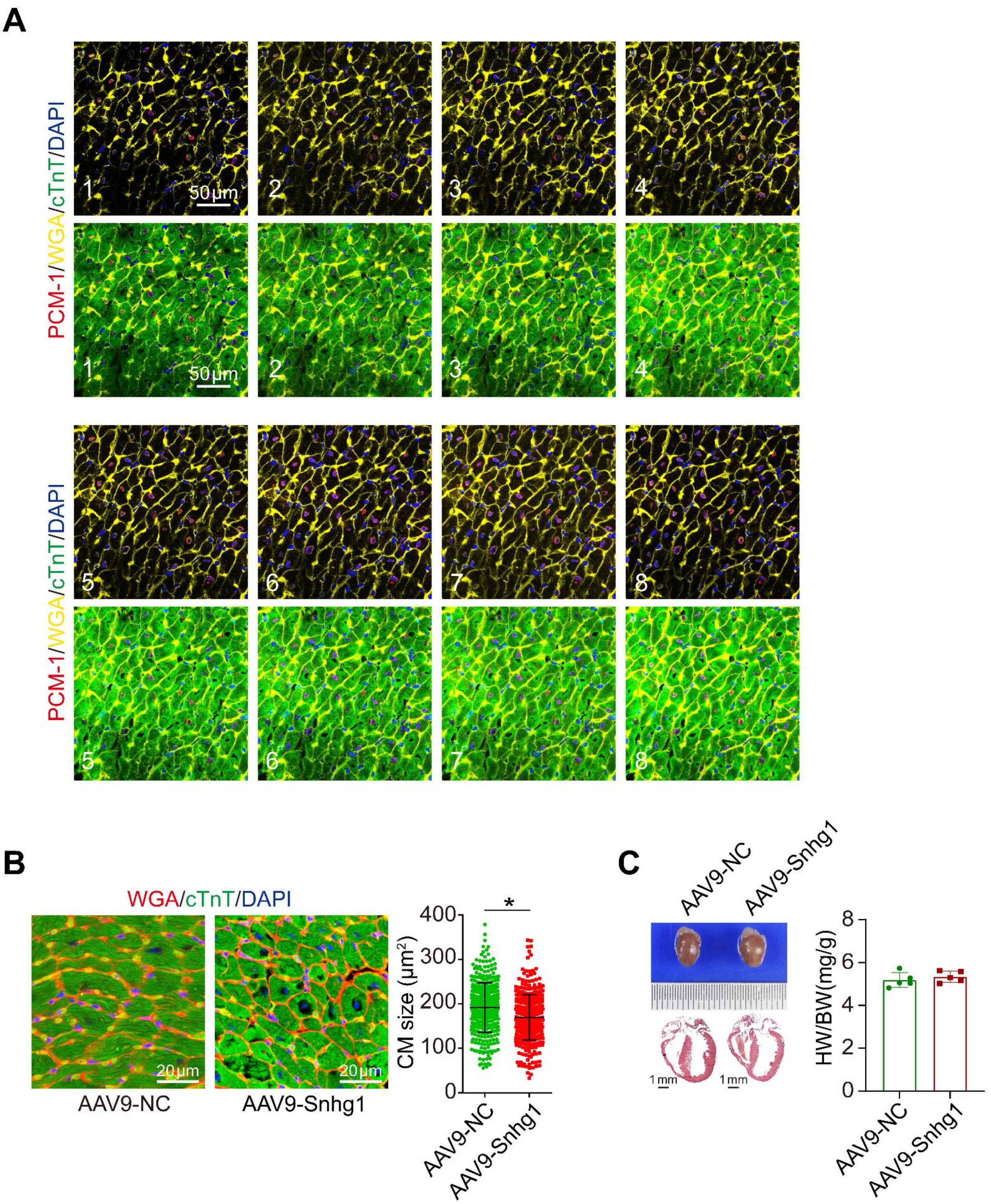
A
PCM-1/WGA/cTnT/DAPI
1
2
3
4
50μm
5
6
7
8
B
WGA/cTnT/DAPI
20μm
AAV9-NC
AAV9-Snhg1
CM size (μm²)
400
300
200
100
0
*
C
HW/BW(mg/g)
8
6
4
2
0
1mm

Figure S7

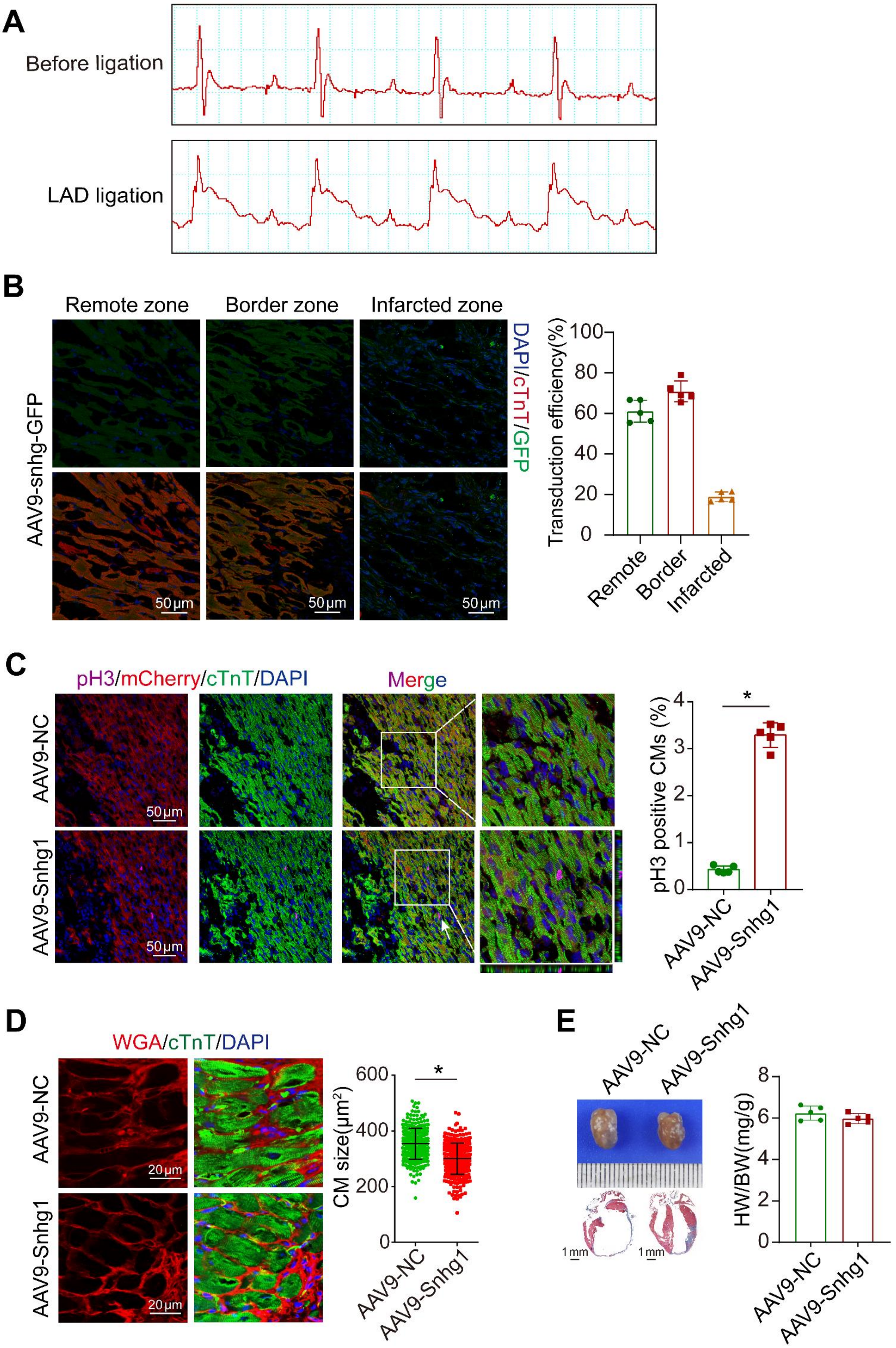
A
Before ligation
LAD ligation
B
Remote zone
Border zone
Infarcted zone
AAV9-snhg-GFP
DAPI/cTnT/GFP
50 μm
Transduction efficiency(%)
0
20
40
60
80
100
Remote
Border
Infarcted
C
pH3/mCherry/cTnT/DAPI
Merge
AAV9-NC
AAV9-Snhg1
50 μm
pH3 positive CMs (%)
0
1
2
3
4
*
D
WGA/cTnT/DAPI
AAV9-NC
AAV9-Snhg1
20 μm
CM size(μm²)
0
200
400
600
*
E
AAV9-NC
AAV9-Snhg1
1 mm
HW/BW(mg/g)
0
2
4
6
8

Figure S8

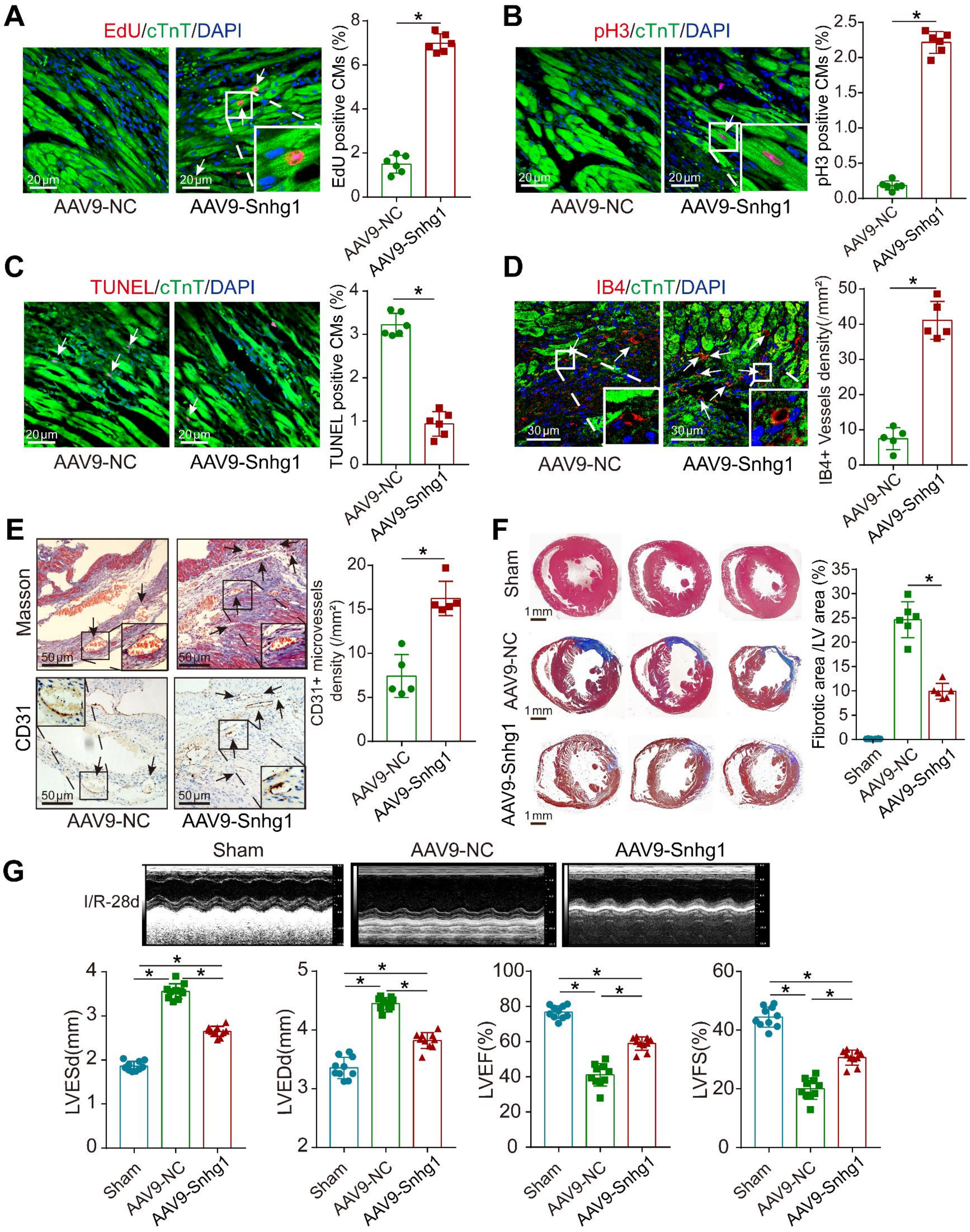
A
EdU/cTnT/DAPI
20μm
AAV9-NC
AAV9-Snhg1
EdU positive CMs (%)
B
pH3/cTnT/DAPI
pH3 positive CMs (%)
C
TUNEL/cTnT/DAPI
TUNEL positive CMs (%)
D
IB4/cTnT/DAPI
30μm
IB4+ Vessels density(/mm²)
E
Masson
CD31
50μm
CD31+ microvessels density (/mm²)
F
Sham
1mm
Fibrotic area /LV area (%)
G
I/R-28d
LVESd(mm)
LVEDd(mm)
LVEF(%)
LVFS(%)

Figure S9

A

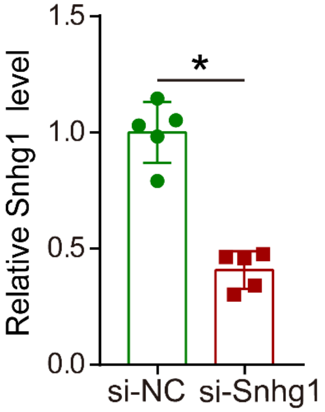

B

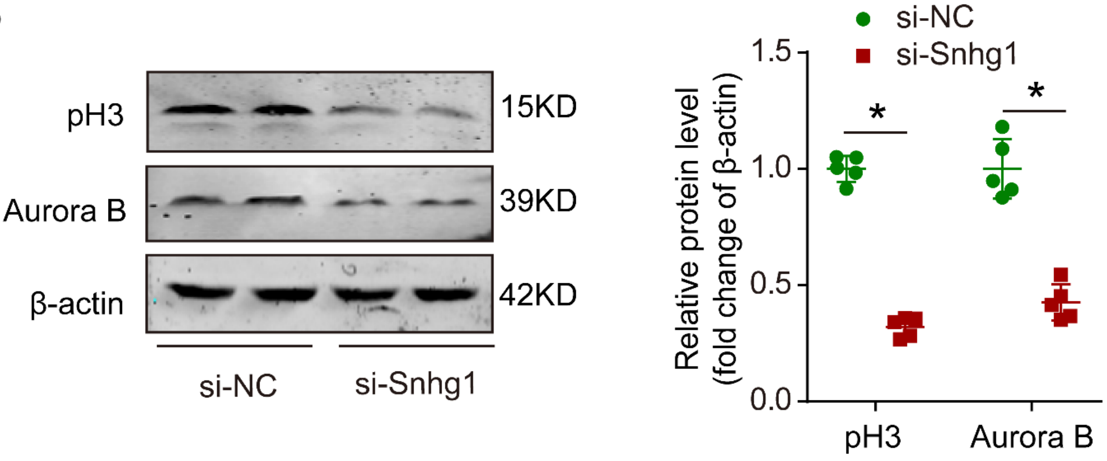

Figure S10

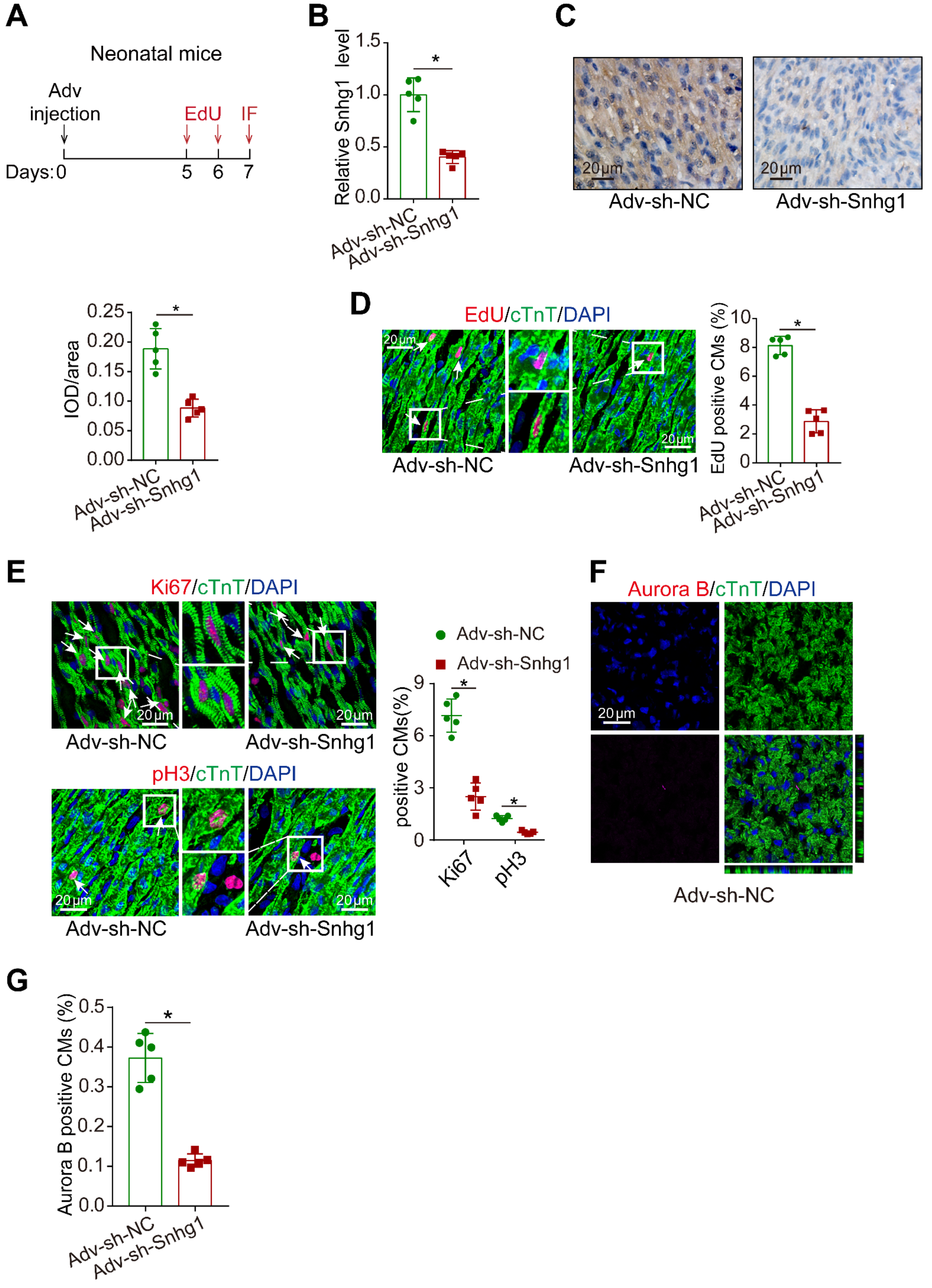
A
Neonatal mice
Adv
injection
EdU
IF
Days:0
5
6
7
B
Relative Snhg1 level
1.5
1.0
0.5
0.0
*
Adv-sh-NC
Adv-sh-Snhg1
C
20 μm
20 μm
Adv-sh-NC
Adv-sh-Snhg1
IOD/area
0.25
0.20
0.15
0.10
0.05
0.00
*
Adv-sh-NC
Adv-sh-Snhg1
D
EdU/cTnT/DAPI
20 μm
20 μm
Adv-sh-NC
Adv-sh-Snhg1
EdU positive CMs (%)
10
8
6
4
2
0
*
Adv-sh-NC
Adv-sh-Snhg1
E
Ki67/cTnT/DAPI
20 μm
20 μm
Adv-sh-NC
Adv-sh-Snhg1
pH3/cTnT/DAPI
20 μm
20 μm
Adv-sh-NC
Adv-sh-Snhg1
Adv-sh-NC
Adv-sh-Snhg1
positive CMs(%)
9
6
3
0
*
*
Ki67
pH3
F
Aurora B/cTnT/DAPI
20 μm
Adv-sh-NC
G
Aurora B positive CMs (%)
0.5
0.4
0.3
0.2
0.1
0.0
*
Adv-sh-NC
Adv-sh-Snhg1

Figure S11

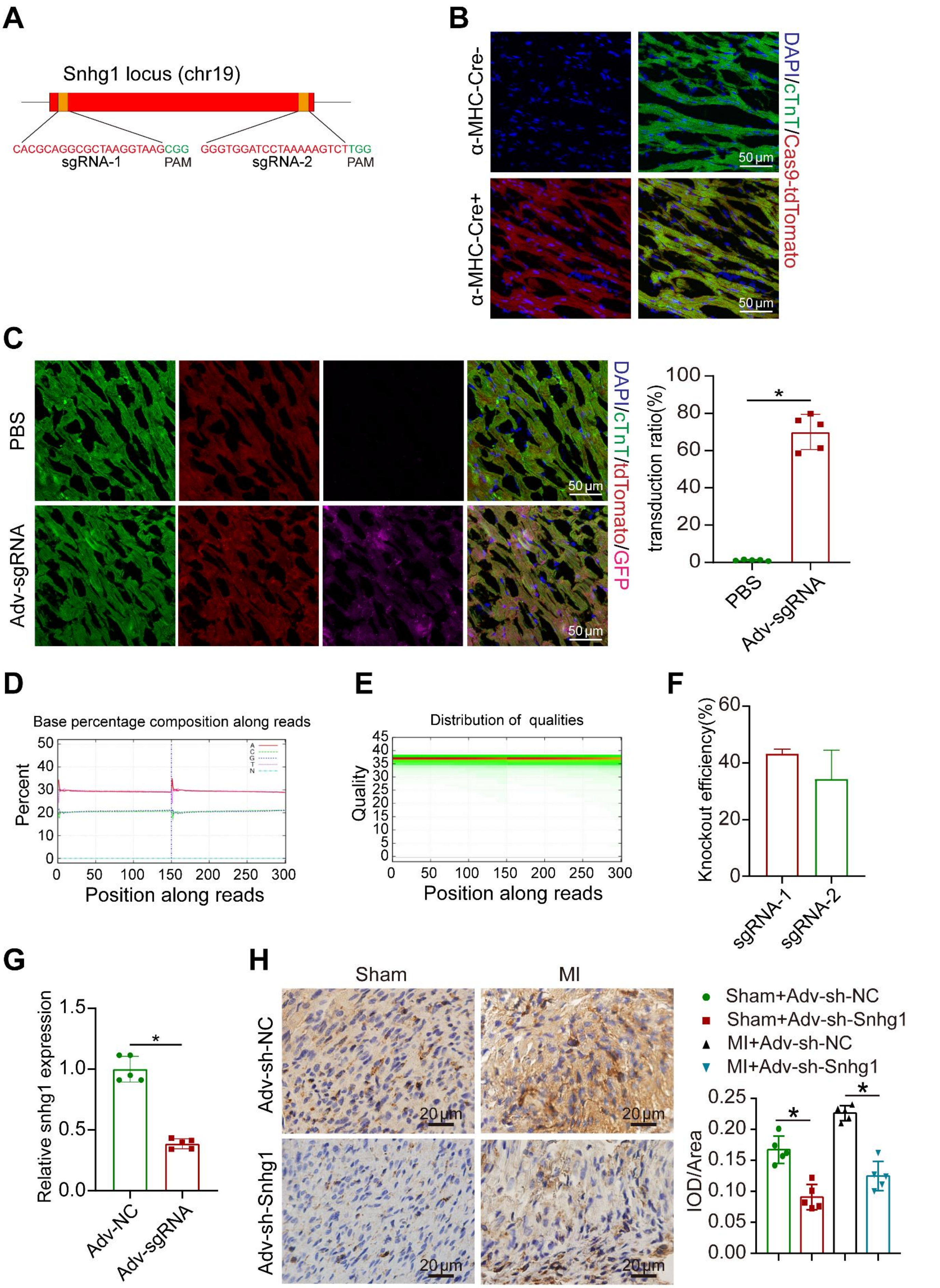
A
Snhg1 locus (chr19)
CACGCAGGCGCTAAGGTAAGCGG
sgRNA-1
PAM
GGGTGGATCCTAAAAAGTCTTGG
sgRNA-2
PAM
B
α-MHC-Cre-
α-MHC-Cre+
DAPI/cTnT/Cas9-tdTomato
50 μm
C
PBS
Adv-sgRNA
DAPI/cTnT/tdTomato/GFP
50 μm
transduction ratio(%)
*
D
Base percentage composition along reads
Percent
Position along reads
E
Distribution of qualities
Quality
Position along reads
F
Knockout efficiency(%)
sgRNA-1
sgRNA-2
G
Relative snhg1 expression
Adv-NC
Adv-sgRNA
H
Sham
MI
Adv-sh-NC
Adv-sh-Snhg1
20 μm
Sham+Adv-sh-NC
Sham+Adv-sh-Snhg1
MI+Adv-sh-NC
MI+Adv-sh-Snhg1
IOD/Area

Figure S12

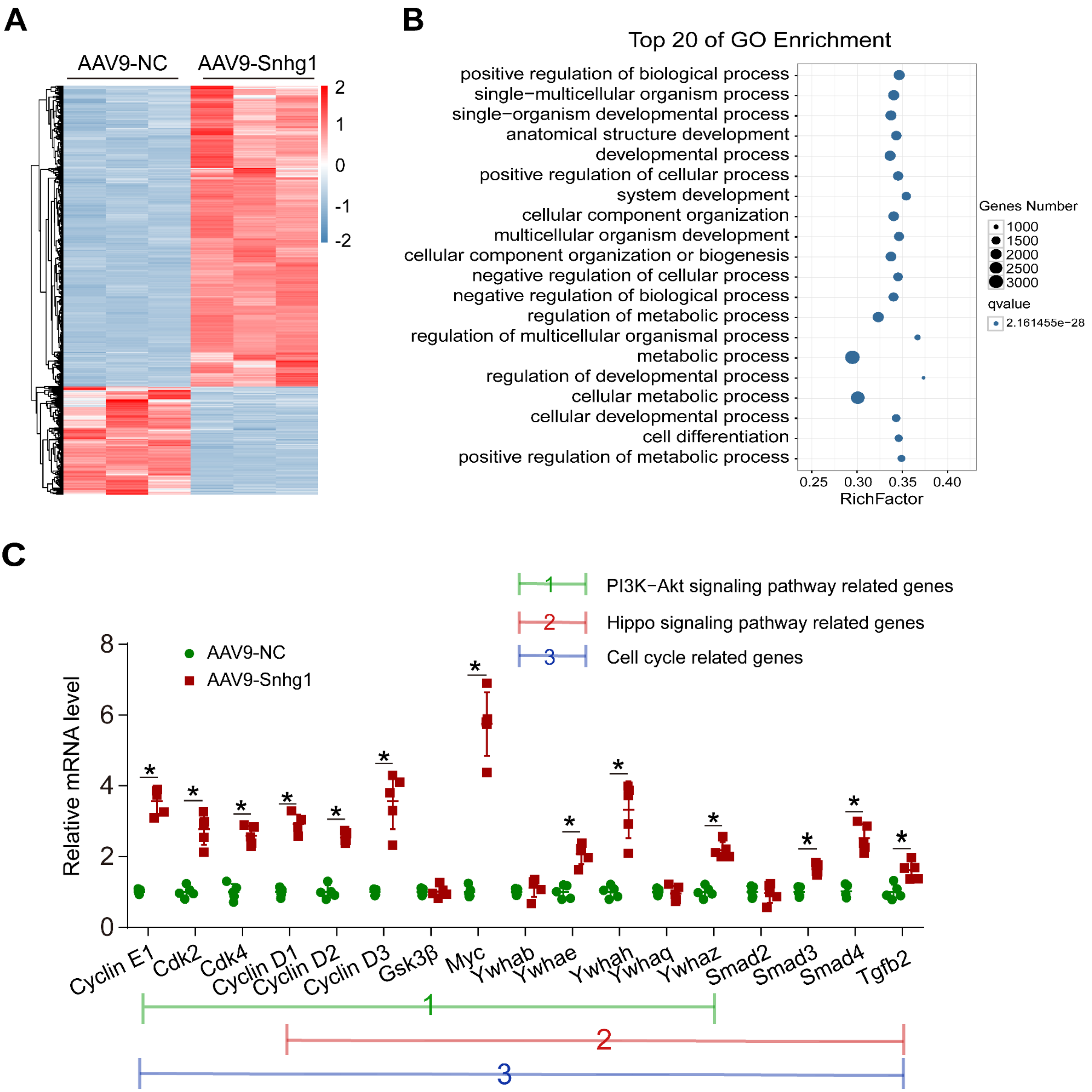
A
AAV9-NC
AAV9-Snhg1
2
1
0
-1
-2
B
Top 20 of GO Enrichment
positive regulation of biological process
single−multicellular organism process
single−organism developmental process
anatomical structure development
developmental process
positive regulation of cellular process
system development
cellular component organization
multicellular organism development
cellular component organization or biogenesis
negative regulation of cellular process
negative regulation of biological process
regulation of metabolic process
regulation of multicellular organismal process
metabolic process
regulation of developmental process
cellular metabolic process
cellular developmental process
cell differentiation
positive regulation of metabolic process
0.25
0.30
0.35
0.40
RichFactor
Genes Number
1000
1500
2000
2500
3000
qvalue
2.161455e−28
C
1 PI3K−Akt signaling pathway related genes
2 Hippo signaling pathway related genes
3 Cell cycle related genes
AAV9-NC
AAV9-Snhg1
Relative mRNA level
8
6
4
2
0
Cyclin E1
Cdk2
Cdk4
Cyclin D1
Cyclin D2
Cyclin D3
Gsk3β
Myc
Ywhab
Ywhae
Ywhah
Ywhaq
Ywhaz
Smad2
Smad3
Smad4
Tgfb2
1
2
3

Figure S13

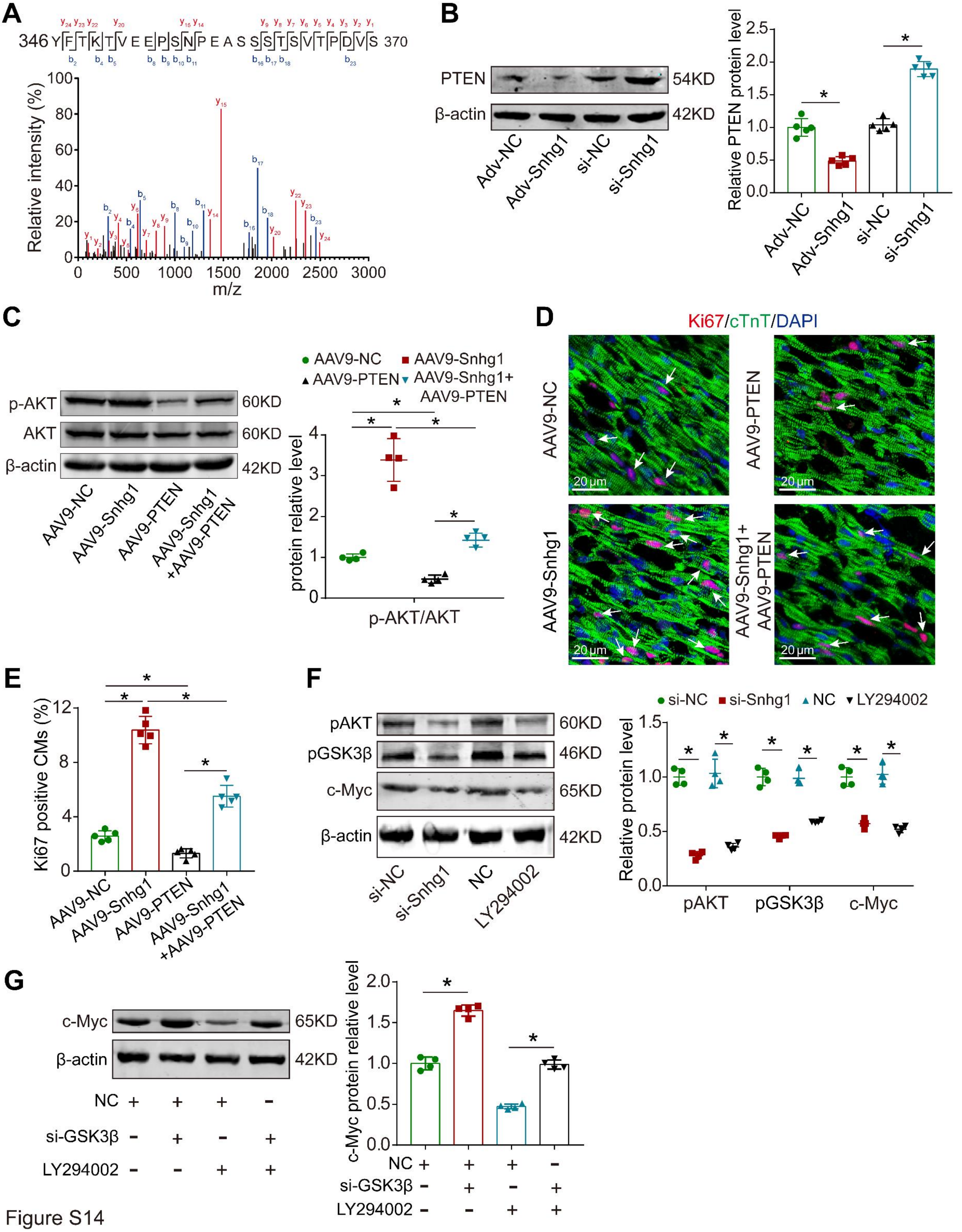

A
346 YFTKTVEEPSNPEASSSTSVTPDVS 370
Relative intensity (%)
m/z
B
PTEN
54KD
β-actin
42KD
Adv-NC
Adv-Snhg1
si-NC
si-Snhg1
Relative PTEN protein level
C
p-AKT
AKT
60KD
β-actin
42KD
AAV9-NC
AAV9-Snhg1
AAV9-PTEN
AAV9-Snhg1 +AAV9-PTEN
AAV9-Snhg1+ AAV9-PTEN
protein relative level
p-AKT/AKT
D
Ki67/cTnT/DAPI
20 μm
E
Ki67 positive CMs (%)
F
pAKT
60KD
pGSK3β
46KD
c-Myc
65KD
si-NC
si-Snhg1
NC
LY294002
Relative protein level
G
si-GSK3β
c-Myc protein relative level

Figure S14

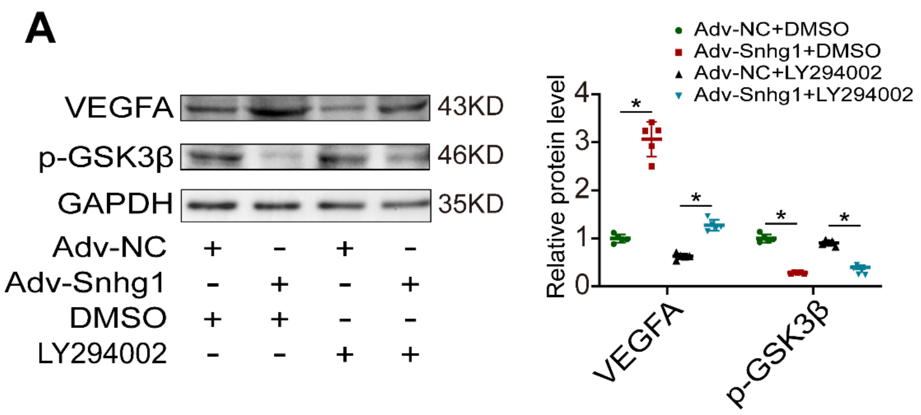
A
VEGFA
p-GSK3β
GAPDH
43KD
46KD
35KD
Adv-NC + - + -
Adv-Snhg1 - + - +
DMSO + + - -
LY294002 - - + +
Adv-NC+DMSO
Adv-Snhg1+DMSO
Adv-NC+LY294002
Adv-Snhg1+LY294002
Relative protein level
0
1
2
3
4
*
VEGFA
p-GSK3β

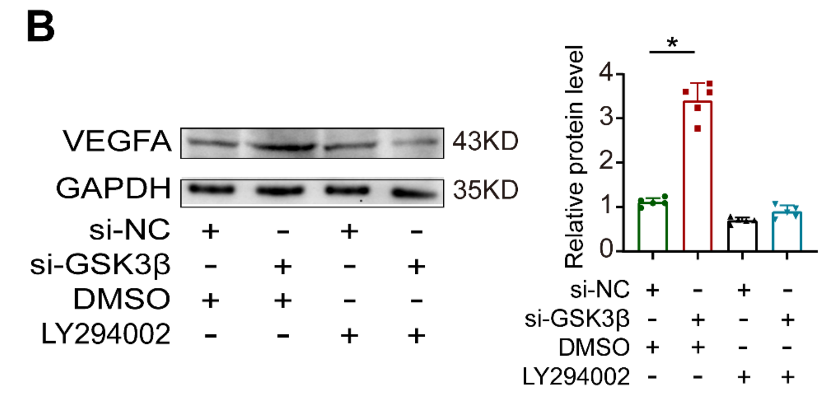
B
VEGFA
GAPDH
43KD
35KD
si-NC + - + -
si-GSK3β - + - +
DMSO + + - -
LY294002 - - + +
Relative protein level
0
1
2
3
4
*
si-NC + - + -
si-GSK3β - + - +
DMSO + + - -
LY294002 - - + +

Figure S15

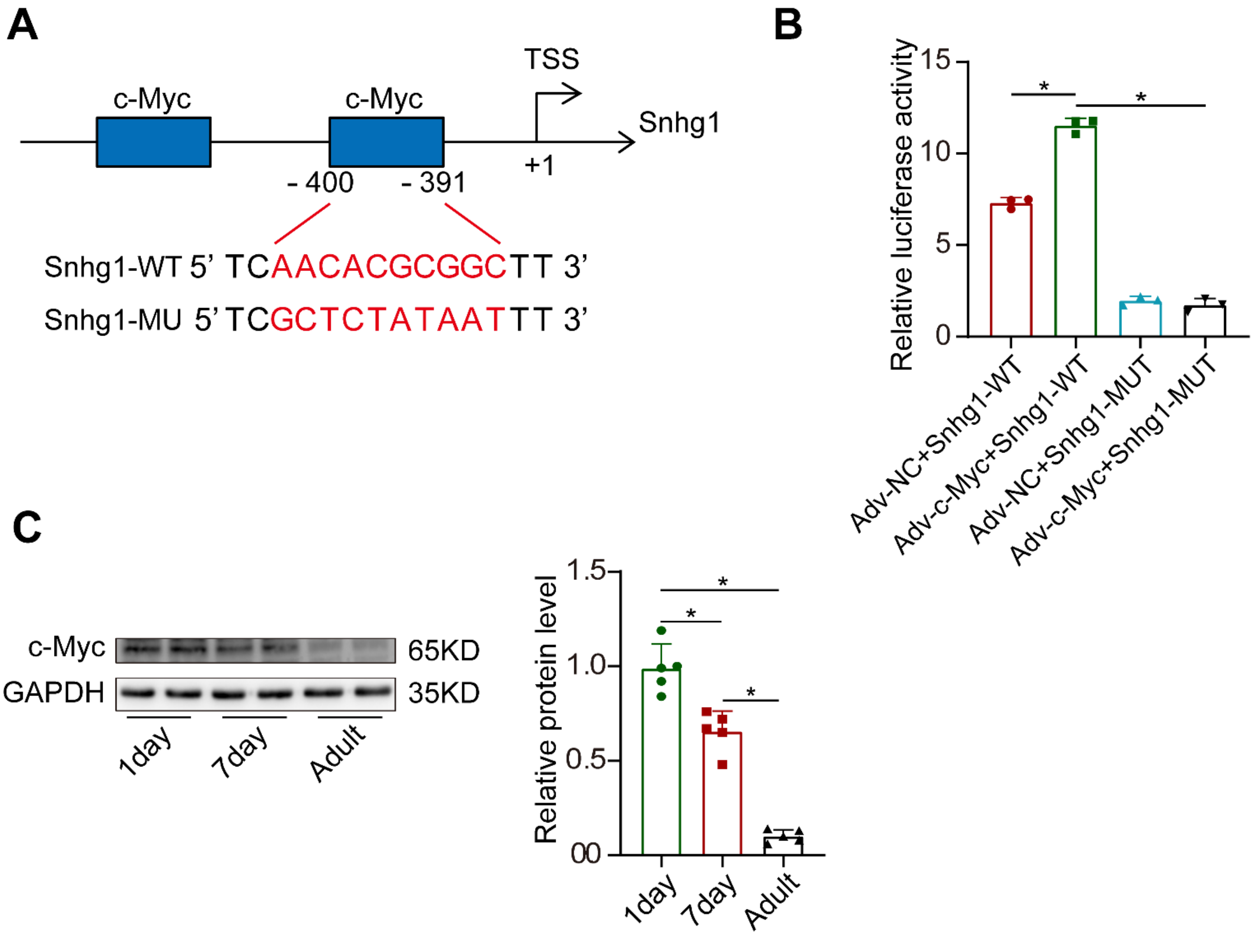
A
c-Myc
c-Myc
TSS
Snhg1
+1
- 400
- 391
Snhg1-WT 5' TCAACACGCGGCTT 3'
Snhg1-MU 5' TCGCTCTATAATTT 3'
B
Relative luciferase activity
15
10
5
0
Adv-NC+Snhg1-WT
Adv-c-Myc+Snhg1-WT
Adv-NC+Snhg1-MUT
Adv-c-Myc+Snhg1-MUT
C
c-Myc
GAPDH
65KD
35KD
1day
7day
Adult
Relative protein level
1.5
1.0
0.5
00
1day
7day
Adult

Figure S16

Supplementary Table 1: Sequences of primers for RT-qPCR

	Forward	Reverse
Snhg1	TTTGCTTGTAGTCAGGGTGCT	AGCCAGACACACCATCTCTG
CCNA2	GCCTTCACCATTCATGTGGAT	TTGCTCCGGGTAAAGAGACAG
CCNB1	CTCAGGGTCACTAGGAACACG	GCTCTTCGCTGACTTTATTACC
CCND2	CCAGACTGTGCCTTGGGAAT	GACACAGGGACAAGTGTGGT
CCNE1	CAGAGCAGCGAGCAGGAGA	GCAGCTGCTTCCACACCACT
CDK2	AGTTGACGGGAGAAGTTGTGG	TGACGATATTAGGGTGATTAAG
CDK4	CCCTCTTCTCACTCTGCGTC	TGCCAGAGATGGAGGAGTCT
CDK6	AGCTGTCTCCACCACCCAC	GGCCATCTGTCGTTAGCCAG
p21	CACAGCTCAGTGGACTGGAA	CCACCACCACACACCATAGA
β-actin	TGCTGTCCCTGTATGCCTCTG	TTGATGTCACGCACGATTTCC

Full unedited gel for Figure 6F

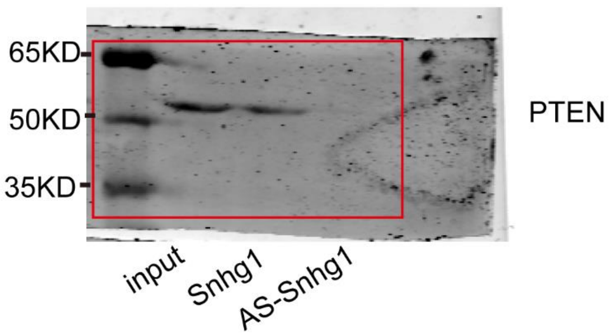

Full unedited gel for Figure 6I

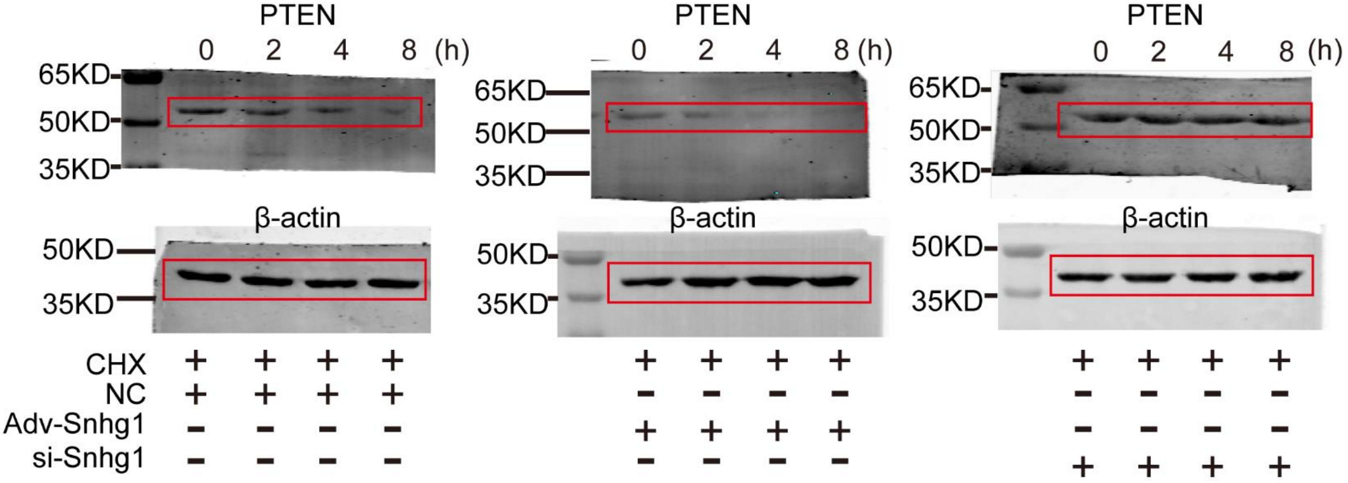

Full unedited gel for Figure 6J

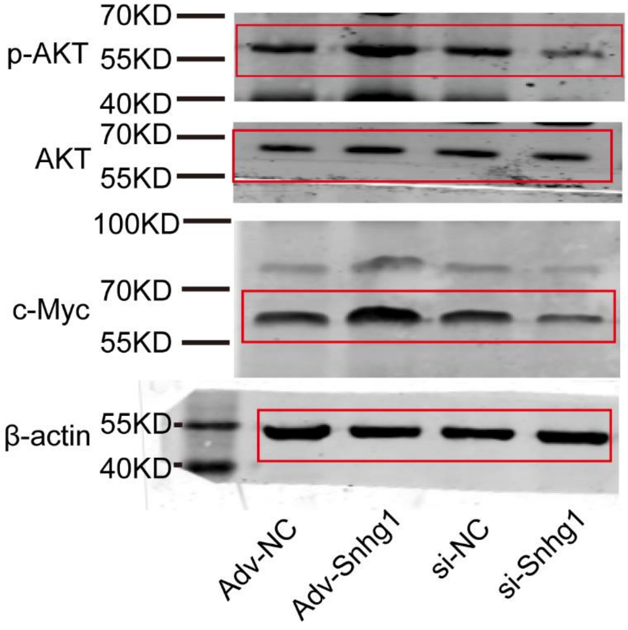

Full unedited gel for Figure 6K

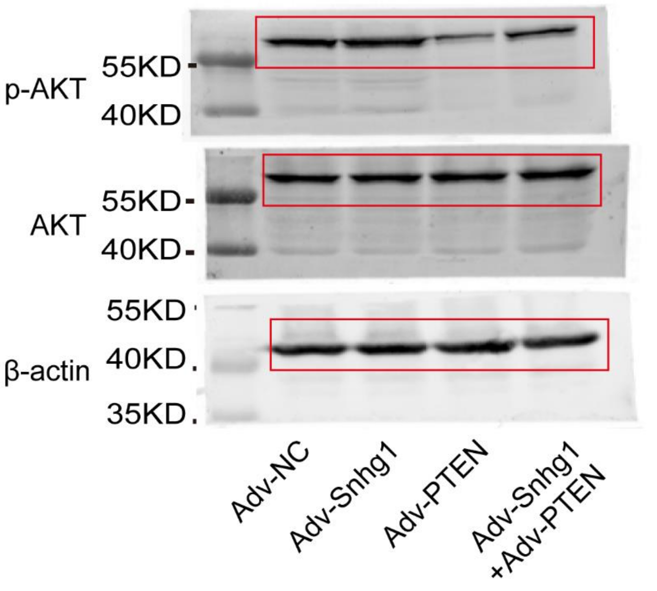

Entire unedited gel for representative cropped gels of Fig.6F,6I,6J,6K. Representative cropped gels are marked by red box.

Full unedited gel for Figure 6M

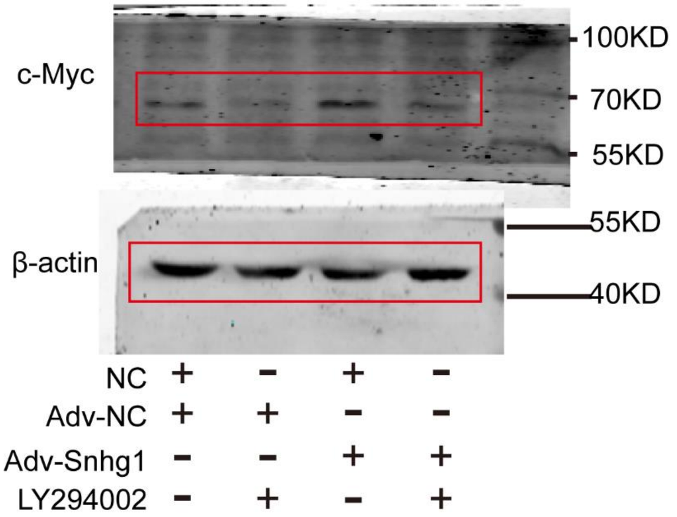

Full unedited gel for Figure 7B

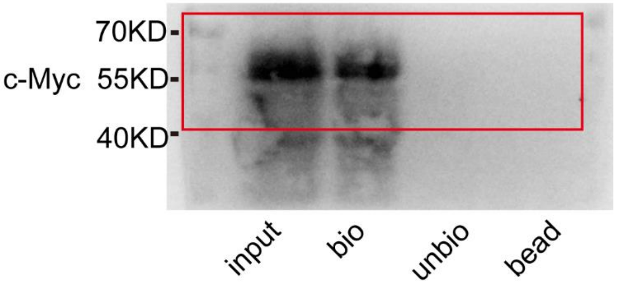

Full unedited gel for Figure 7G

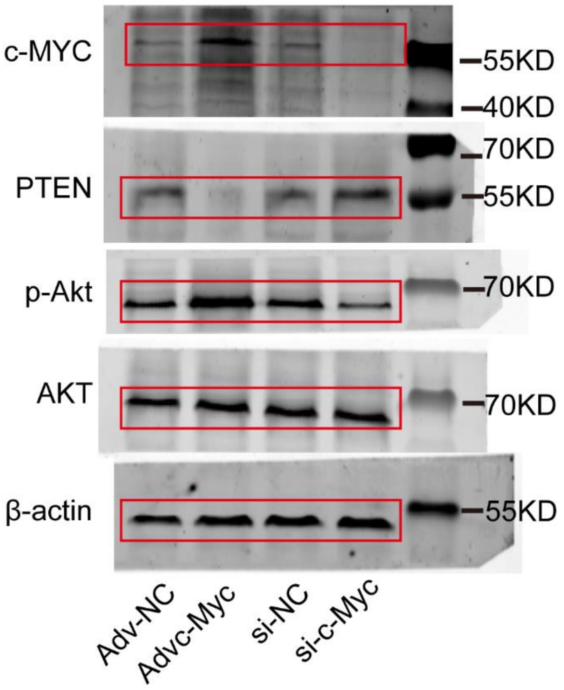

Full unedited gel for Figure 7I

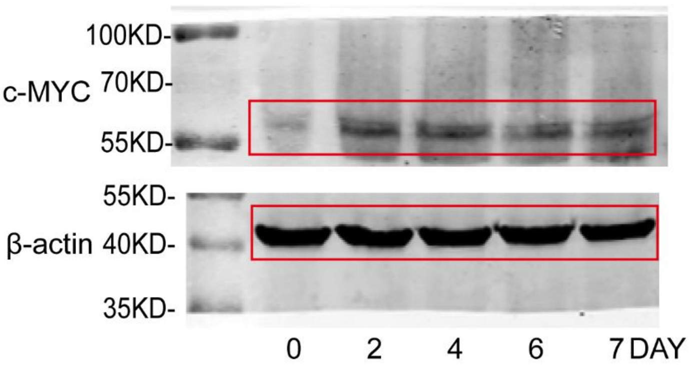

Entire unedited gel for representative cropped gels of Fig.6M,7B,7G,7I. Representative cropped gels are marked by red box.

Full unedited gel for Figure 8C

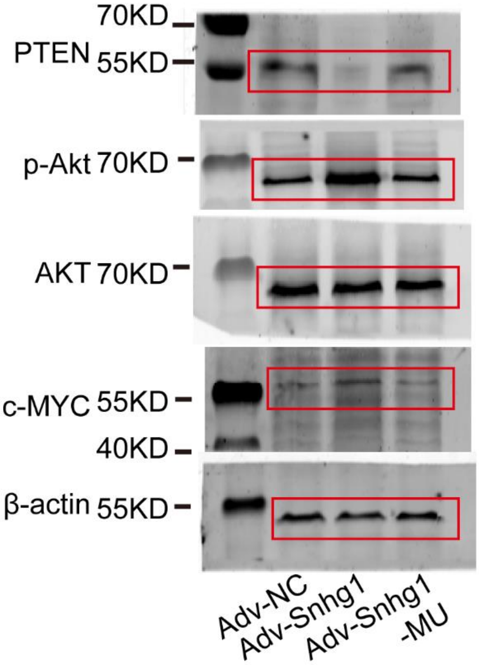

Full unedited gel for Figure S2E

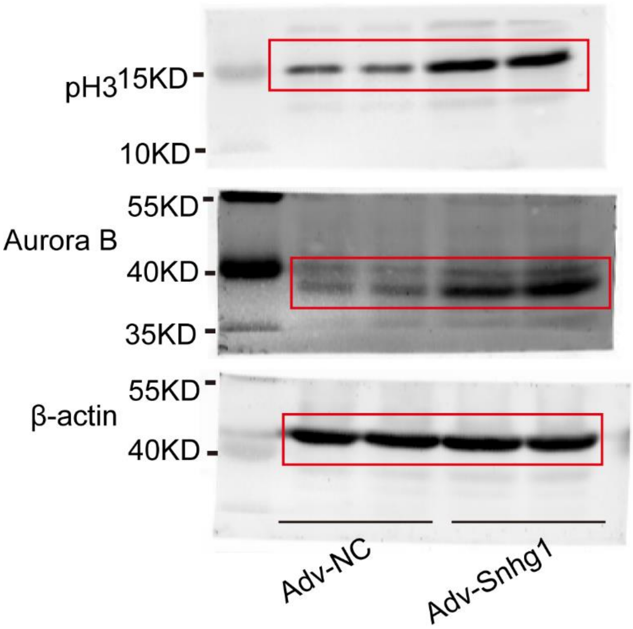

Full unedited gel for Figure S10B

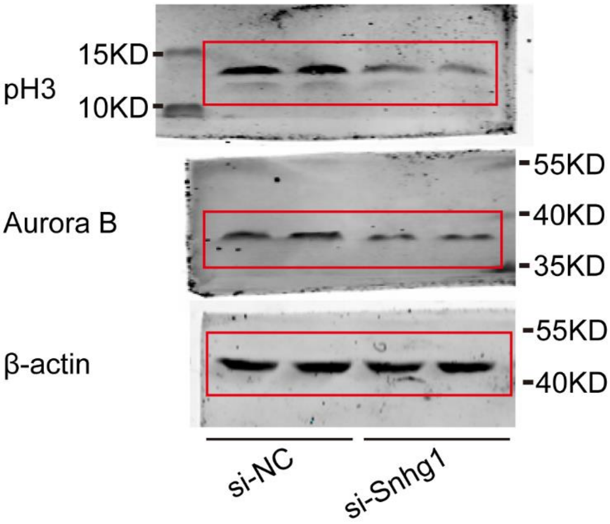

Full unedited gel for Figure S14B

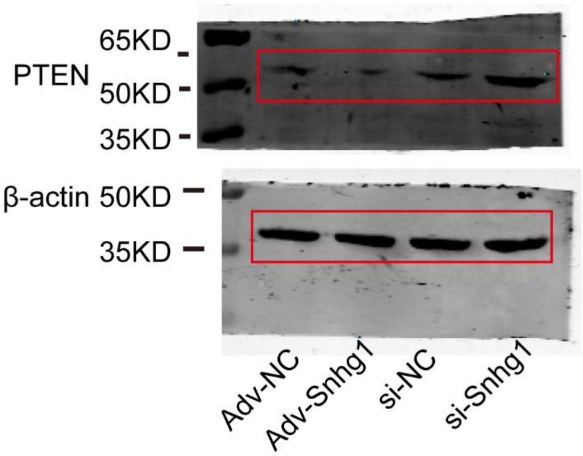

Full unedited gel for Figure S14C

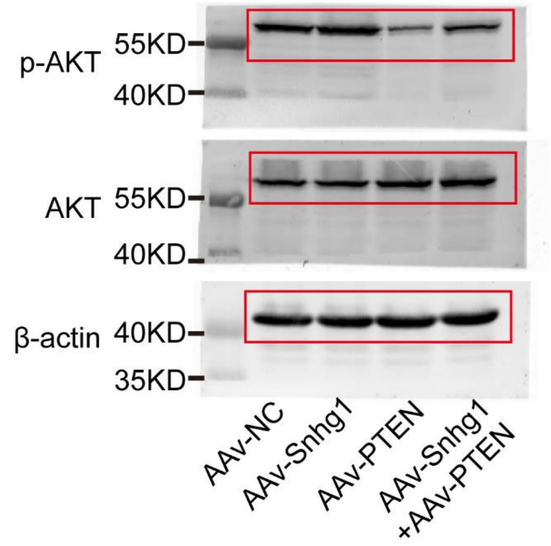

Entire unedited gel for representative cropped gels of Fig.8C,S2E,S10B,S14B,S14C.
Representative cropped gels are marked by red box.

Full unedited gel for Figure S14F

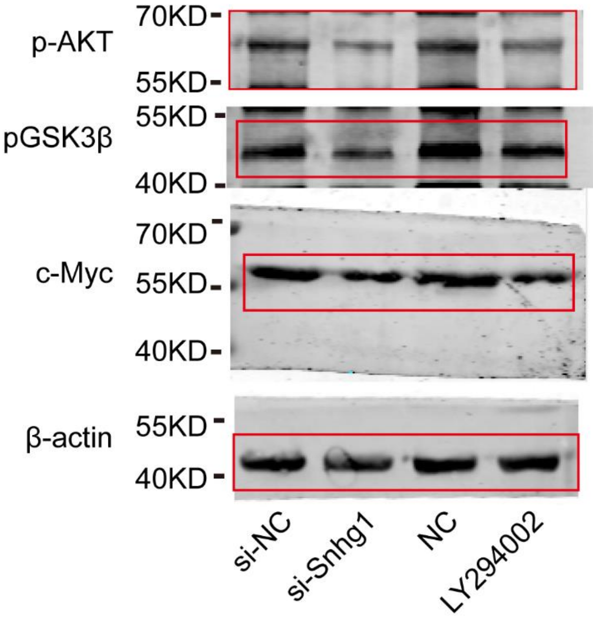

Full unedited gel for Figure S14G

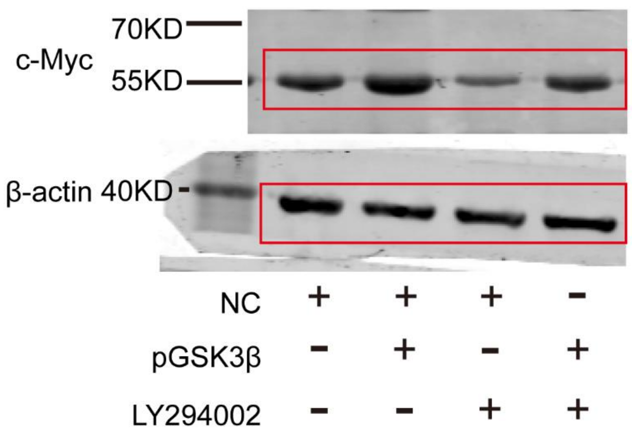

Entire unedited gel for representative cropped gels of Fig.S14F,S14G. Representative cropped gels are marked by red box.
